# Characterization of SMG7 14-3-3-like domain reveals phosphoserine binding-independent regulation of p53 and UPF1

**DOI:** 10.1038/s41598-019-49229-3

**Published:** 2019-09-11

**Authors:** Lauren E. Cowen, Hongwei Luo, Yi Tang

**Affiliations:** Department of Regenerative and Cancer Cell Biology, Albany Medical College, 47 New Scotland Ave., Albany, NY 12208 USA

**Keywords:** Double-strand DNA breaks, RNA quality control

## Abstract

The 14-3-3-related protein SMG7 plays critical roles in regulation of DNA damage response and nonsense-mediated mRNA decay (NMD). Like 14-3-3, SMG7 engages phosphoserine-dependent protein interactions; however, the precise role of phosphorylation-mediated SMG7 binding remains unknown. Here, we show that DNA damage-induced SMG7-p53 binding requires phosphorylated Ser15 on p53, and that substitution of the conserved lysine residue K66 in the SMG7 14-3-3-like domain with the glutamic acid (E) abolishes interactions with its client proteins p53 and UPF1. Unexpectedly, loss of phosphoserine-dependent SMG7 binding does not significantly affect p53 stabilization/activation, and p53-dependent cell growth arrest or apoptosis upon DNA damage. Also surprisingly, cells expressing the SMG7 K66E-knockin mutant retain fully functional UPF1-mediated NMD. These findings are highly unusual, given that phosphorylation-mediated 14-3-3 binding has essential roles in numerous cellular signaling pathways. Thus, our studies suggest that 14-3-3-like proteins such as SMG7 likely function using additional distinct regulatory mechanisms besides phosphoserine-mediated protein interactions.

## Introduction

The 14-3-3 family of proteins is highly conserved, and contains seven members (β, γ, ε, η, σ, τ, and ζ), which typically exist in homo or heterodimeric complexes^1^. 14-3-3 proteins interact with phosphoserine/threonine residues on a plethora of client proteins, acting as a cofactors, chaperones or regulatory factors^2,3^. Through these phosphoserine-mediated interactions, 14-3-3 can modulate the phosphorylation status, cause dimer formation or regulate subcellular localization of its partners. The 14-3-3 proteins regulate numerous stress response pathways including cell cycle control and apoptosis through protein-protein interactions via one of three main binding motifs^4^. For example, in response to DNA damage stress, 14-3-3σ promotes the function of tumor suppressor p53 to initiate cell cycle arrest^5,6^.

The nonsense-mediated mRNA decay pathway (NMD) is a critical mRNA surveillance pathway, which degrades and eliminates aberrant mRNAs containing premature translation termination codons (PTC)^7^. Recent studies have identified Suppressor for Morphological defects of Genitalia 7 (SMG7) as a key factor in NMD and its N-terminal region is structurally homologous to 14-3-3 proteins^8,9^. This 14-3-3-like domain of SMG7 resembles the 14-3-3 crescent shaped binding cleft and contains the critical conserved residue lysine 66 (K66) required for interaction with phosphorylated serine/threonine residues^8^. Furthermore, SMG7 forms a tight heterodimer with the NMD factor and 14-3-3-like protein SMG5 similar to 14-3-3 family members^10^. Canonically, NMD is regulated primarily through phosphorylation of NMD master regulator UPF1^11–13^. SMG1 kinase phosphorylates UPF1 at Ser1096, which recruits the SMG7/SMG5 heterodimer to target mRNAs via interaction with SMG7’s 14-3-3-like domain^14,15^. Recruitment of phosphatase PP2A by the SMG7/SMG5 complex facilitates dephosphorylation of UPF1, leading to a cycle of UPF1 phosphorylation and dephosphorylation which is critical for NMD^12^. Besides S1096, UPF1 can be phosphorylated on many other sites, and hyperphosphorylation of UPF1 has been shown to be important for its full function in NMD in a recent study^16^.

p53, a potent inhibitor of cell growth and survival, is tightly regulated by the E3 ligase MDM2, which ubiquitinates and degrades p53, keeping p53 at low levels and allowing rapid response to cell stress^17,18^. In our recent study, we identified SMG7 as a critical regulator for p53 stability and p53-mediated DNA damage response^19^. SMG7 interacts with MDM2 and promotes an inhibitory phosphorylation of MDM2 at Ser395 by ATM, which leads to p53 stabilization^19,20^. Moreover, SMG7 also binds p53 and this interaction appears highly dependent on DNA damage, suggesting that SMG7 may directly regulate p53^19^. Beside regulating p53 stability, SMG7 functions via UPF1-mediated NMD to control the expression level of p53β, an alternatively spliced p53 isoform that is critically involved in cell cycle regulation and stress response^21–25^. Thus, our studies demonstrate that SMG7 regulates two distinct cellular functions – DNA damage response and nonsense-mediated mRNA decay. As the 14-3-3-like domain of SMG7 is required for binding to UPF1 and may likely be involved in mediating DNA damage-induced interaction with p53, we investigated the role of SMG7 binding in the regulation of p53 and UPF1.

In the present study, we examined the DNA damage-induced SMG7 interaction with p53, and identified the SMG7-binding motif shared by its client proteins UPF1 and p53. In assessing the role of phosphorylation-mediated SMG7 binding to p53 and UPF1, we provide direct evidence to show a more complex SMG7 regulation of p53-mediated DNA damage response and UPF1-dependent NMD.

## Results

### Phospho-Ser15 of p53 is required for interaction with SMG7 upon DNA damage

**Figure 1:**
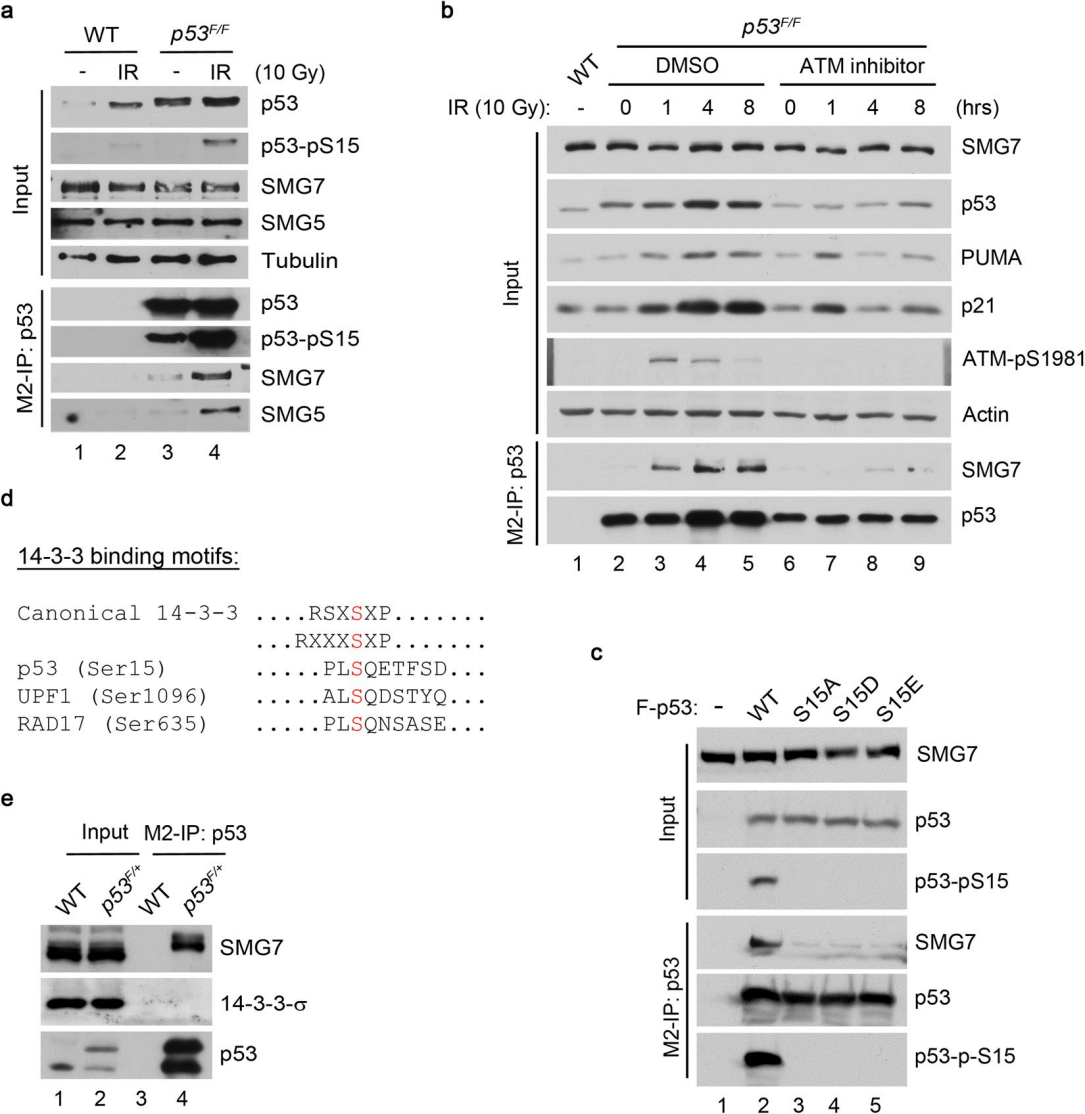
Phospho-Ser15 of p53 is required for interaction with SMG7 upon DNA damage. (**a**) Cell extracts from HCT116 cells (wild type or endogenously flag tagged p53) were irradiated and the corresponding α-Flag (p53) immunoprecipitates were analyzed by western blot using antibodies as indicated. (**b**) Control or irradiated cells treated with or without ATM inhibitor KU55933 were analyzed as in (**a)**. (**c**) Cell extracts from H1299 cells co-transfected with SMG7 and F-p53 constructs. The α-Flag (p53) immunoprecipitates were analyzed by western blot using antibodies as indicated. (**d**) Alignment of SMG7-binding motif from its client proteins (p53, UPF1 & Rad17) compared to the canonical 14-3-3 binding motifs. Red S represents the critical serine residue that facilitates the interaction. (**e**) Cells treated with etoposide (20 μM, 4 hours) were assayed as in (**a**).

Previous studies show that SMG7 enhances p53 stability and inhibits cell cycle progression after treatment with various DNA-damaging agents including ionizing radiation (IR) and doxorubicin (Doxo)^19^. Interestingly, DNA damage induces a robust interaction of SMG7 with p53, raising the question of how SMG7 binding is regulated and whether it plays a role in p53 stabilization? To address these issues, we utilized HCT116-p53^F/F^ cells expressing endogenous Flag-tagged p53 (F-p53)^26^ and performed p53 immunoprecipitations using anti-Flag antibodies (hereinafter referred to as M2-IP). As expected, IR enhanced interactions between p53 and SMG7, which correlates with the elevated levels of Ser15-phosphorylated p53 (Fig. 1a, lane 3 vs 4, and S1a). The induced SMG7-p53 interaction is not a result of p53 stabilization, as the MDM2 inhibitor Nutlin increased p53 levels but had no effect on SMG7 binding (Supplementary Fig. S1b)^27^.

These results prompted us to assess the role of ATM kinase and its phosphorylation of p53 Ser15 in regulating the p53/SMG7 interaction. Indeed, we found that treatment with the ATM specific inhibitor KU55933 impaired IR-induced p53 interaction with SMG7 as well as activation of p53 target genes *p21* and *PUMA* (Fig. 1b, lanes 3–5 vs 7–9)^28^. To interrogate the role of p53 Ser15 phosphorylation further, we treated cells with the DNA damaging drug etoposide to activate ATM and ATR (ATM and RAD3-related) kinases, both of which phosphorylate p53 at Ser15^29–31^. While inhibition of ATM exhibited no effect on etoposide-induced p53 Ser15 phosphorylation and SMG7 binding as expected, treatment with caffeine, which inhibits both ATM and ATR^32,33^, abolished the interaction between p53 and SMG7 (Supplemental Fig. S1c,d). Given that SMG7 contains a 14-3-3-like domain, these results support the notion that p53 Ser15 phosphorylation may have a direct role in mediating SMG7 interaction. To test this hypothesis directly, we performed immunoprecipitation assays to examine SMG7 binding to wild type or phosphorylation-deficient mutant p53 (S15A, S15D or S15E). Notably, while wild type p53, which is highly phosphorylated at Ser15 when expressed in the cells, binds SMG7 strongly, all three mutations abrogated SMG7-binding activities (Fig. 1c, lane 2 vs 3–5). The inability of phosphomimetic p53 mutant S15D or S15E to bind SMG7 indicates a stringent conformational requirement imposed by phosphoserine for SMG7 binding. To further corroborate these findings, we performed p53 M2-IP followed by treatment with λ phosphatase to remove phosphorylation from p53, and found that when treated with the protein phosphatase, the interaction with SMG7 is strongly reduced (Supplemental Fig. S1e). Taken together, our data suggest that p53 Ser15 phosphorylation by ATM and/or ATR mediates the p53 interaction with SMG7 under various DNA damage conditions.

### Sequence analysis reveals a previously unappreciated binding motif for SMG7

14-3-3 binds phosphoserine/threonine residues within specific motifs present in its client proteins^2^. Studies from our laboratory and others have identified several phosphoserine-dependent SMG7-interacting proteins including UPF1^12–14^, p53 and RAD17 (Ser635, manuscript under review). Interestingly, sequence comparison revealed a previously unknown SQ-containing motif required for SMG7 binding, which is different from the known 14-3-3-binding motifs (Fig. 1d). The finding that DNA damage enhanced the p53-SMG7 interaction but had no effect on p53 association with 14-3-3 further ascertained the distinct nature of the binding motifs for 14-3-3 and SMG7 (Fig. 1e). It is important to note that ATM/ATR phosphorylate the SQ sites of p53 and RAD17^30,31,34^ and SMG1, an ATM-related kinase, phosphorylates UPF1 at Ser1096^35^. Thus, the invariant LSQ sequence surrounded by similar amino acids may constitute a *bona fide* SMG7-binding motif.

### 14-3-3-like domain of SMG7 mediates its interaction with Ser15-phosphorylated p53

**Figure 2:**
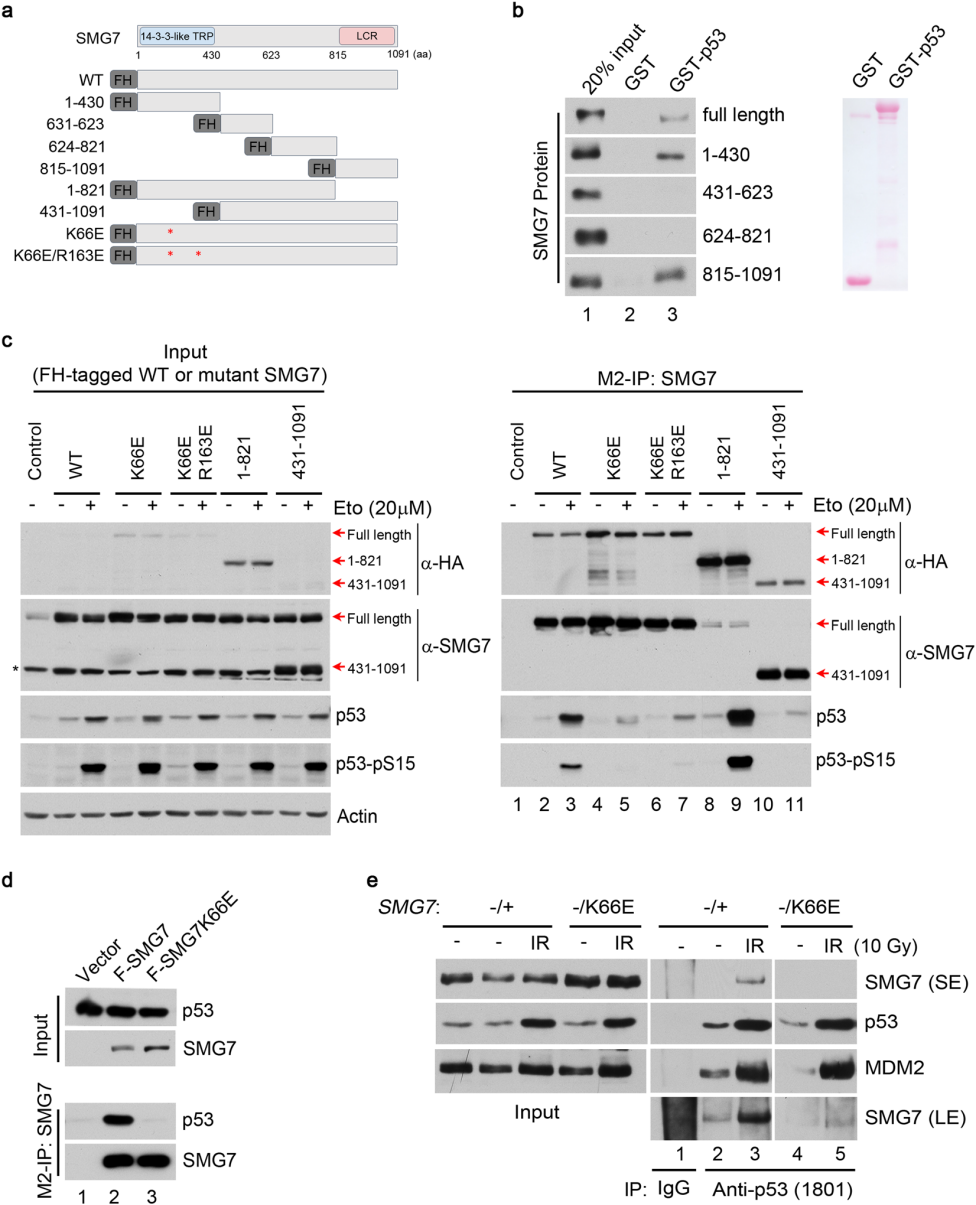
SMG7 14-3-3-like domain mediates its interaction with Ser15-phosphorylated p53. (**a**) Schematic illustrating various SMG7 fragments and point mutants used in (**b**,**c**). FH represents a Flag and HA tag at the 5′ end of all constructs. (**b**) p53 binding to SMG7 *in vitro*. GST or GST-p53 fusion proteins were used in pulldown assays with purified FH-SMG7 (full length or fragments). (**c**) Cell extracts and α-Flag (SMG7) immunoprecipitates from U2OS cells stably expressing FH-SMG7 or mutants treated with etoposide (20 µM, 6 hours) were analyzed by western blot using antibodies as indicated. Red arrows denote various SMG7 fragment expression sizes which were identified using SMG7 and HA antibodies. (**d**) H1299 cells co-transfected with p53 and F-SMG7 or F-SMG7-K66E constructs were analyzed as in (**c**). (**e**) Cell extracts and α-p53 immunoprecipitates from control and irradiated cells were analyzed by western blot using antibodies as indicated (long and short exposures shown).

So far, our data suggest that SMG7’s 14-3-3-like domain may mediate phosphoserine-dependent interaction with p53 under DNA damage conditions. To test this idea, we first mapped p53-binding domains, and found that both SMG7’s N- and C-terminal fragments (1–430aa and 815–1091aa, respectively) can bind p53 *in vitro* (Fig. 2a,b). As GST-p53 purified from bacteria is not phosphorylated on S15, these data suggest that the N-terminal 14-3-3-like domain or C-terminal region of SMG7 may have the potential in p53 binding *in vitro* in a phosphorylation independent manner. This potentially suggests an additional role for the SMG7/p53 interaction possibly via p53 C-terminal region (290–393aa), independent of S15 phosphorylation^19^. However, when the interaction is examined in cells stably expressing full-length or truncated FH-SMG7 (Fig. 2a), only the N-terminal region containing the 14-3-3-like domain is required for SMG7 interaction with Ser15-phosphorylated p53 upon DNA damage (Fig. 2c, lane 9 vs 11). Taken together, our data support our hypothesis that the interaction between p53 and SMG7’s 14-3-3 domain is through the phosphorylated serine 15 residue. This does not exclude the possibility, however, that another phosphorylation independent interaction could also be occurring between p53 and SMG7. As shown previously, SMG7 14-3-3-like domain contains two conserved residues K66 and R163, which are critical for mediating interaction with S1096-phosphorylated UPF1^8,10^. Consistent with these studies, a single amino acid substitution (K66E) abrogated SMG7 interaction with p53, an effect that was not exacerbated by the second mutation R163E (Fig. 2c, lane 3 vs 5 and 7). Furthermore, when co-expressed with p53 in cells, SMG7-K66E failed to interact with Ser15-phosphorylated p53 (Figs 1c and 2d, lane 2 vs 3), indicating that an intact 14-3-3-like domain is indeed essential for phosphoserine-mediated SMG7-p53 interaction.

It is worth noting that K66 of SMG7 is absent in two other closely related 14-3-3-like proteins SMG5 and SMG6, despite that all three have very similar domains and other key conserved residues (Supplementary Fig. S2a)^8^. Interestingly, however, we observed robust SMG5-p53 interactions after IR or treatment with DNA damaging agent camptothecin (CPT) (Fig. 1a and Supplementary Fig. S2b), raising the question whether SMG5 interacts with p53 in a similar fashion as SMG7. To address this issue, we exogenously expressed p53 with the Flag-tagged or untagged SMG5 or SMG7 and performed M2-IP to assess protein complex formation. Intriguingly, we observed very weak association of SMG5 with p53, which was dramatically enhanced by co-expression of wild type SMG7 but not by K66E mutant (Supplementary Fig. S2c,d). On the other hand, overexpression SMG5 had no effect on the SMG7 interaction with p53 (Supplementary Fig. S2c). Thus, these data suggest that the SMG7-SMG5 heterodimer binds p53 via SMG7, and SMG5 is not required for the maximum p53 interaction. Moreover, the fact that SMG5 lacks K66 highlights the critical nature of this conserved residue with respect to phosphoserine-mediated p53 binding and serves as a unique distinction for the 14-3-3-like protein SMG7 in regulation of p53.

### Stressed-induced SMG7 binding is not essential for p53-mediated DNA damage response

**Figure 3:**
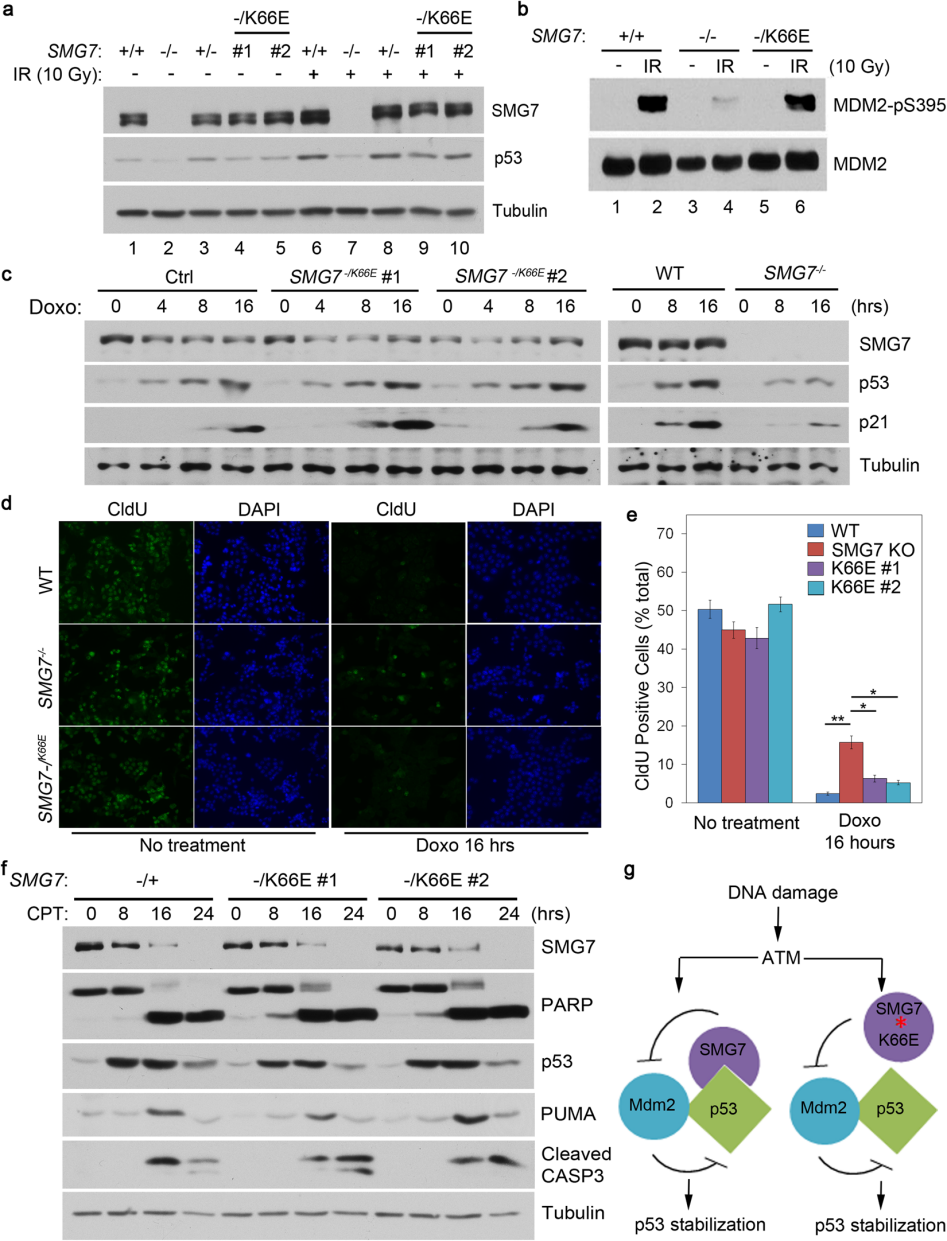
Disruption of stressed-induced SMG7 binding to p53 does not significantly affect p53 stabilization, activation or DNA damage response. (**a**) Western blot analysis of cell extracts from control and irradiated (6 hours) cells of various *SMG7* genotypes. (**b**) α-MDM2 immunoprecipitates from control or irradiated cells were assayed for Ser395 phosphorylation. (**c**) Cell extracts from control or Doxo-treated (0.4 μM) cells were analyzed by western blot using antibodies as indicated. (**d**,**e**) Control or Doxo-treated (0.4 μM, 16 hours) cells were fed with 10 μM CldU for 1 hour and then processed for CldU (green) and DAPI (blue) staining, followed by fluorescence microscopy. Representative images were shown in (**d**), and quantification of CldU-positive cells from five independent experiments in (**e**). (*p < 0.05, **p < 0.005; error bars represent SEM). (**f**) Western blot analysis of cell extracts from control or CPT-treated (1 µM) cells using antibodies as indicated. (**g**) A working model for SMG7 regulation of p53 stability upon DNA damage.

To investigate the role of phosphorylation-mediated SMG7 binding in regulation of p53, we established HCT116 *SMG7 *K66E knockin cell lines via AAV-mediated homologous recombination^26,36^, a gene targeting approach we previously utilized to generate *SMG7* knockout (KO) cells (Supplementary Fig. S3)^19^. Assessment of these cell lines showed that the K66E mutation had no effect on the protein levels of p53 and SMG7 (Figs 2e and 3a,c). Importantly, the IR-induced interaction between p53 and SMG7 was abolished in K66E mutant cells, whereas interaction of p53 with Mdm2 remained unaffected, confirming the specific effect of K66E on SMG7-p53 binding (Fig. 2e, lane 3 vs 5). Then we examined IR-induced p53 stabilization, and found that p53 induction is impaired in *SMG7* KO cells; surprisingly, the K66E mutant cells did not show any major defect in p53 stabilization (Fig. 3a and Supplementary Fig. S4a). Moreover, we made similar observations in cells treated with two other DNA-damaging drugs Doxo and CPT (Supplementary Fig. S4b,c). p53 stabilization upon DNA damage requires ATM phosphorylation of MDM2, which inhibits MDM2 E3 ligase activity^20^. Consistent with these results, IR indeed induced similar levels of Ser395-phosphorlated MDM2 in both wild type and K66E mutant cells, as opposed to *SMG7* KO cells (Fig. 3b, lane 4 vs 2 and 6). Furthermore, the interaction between SMG7 and MDM2 is retained in the presence of the K66E mutation (Supplementary Fig. S4d). These data suggest that the SMG7-K66E fully retains the ability to inhibit MDM2 after DNA damage (Fig. 3g), and phosphoserine-mediated SMG7 binding to p53 is not essential for the p53 stabilization.

Activation of p53 following DNA damage induces expression of its target genes *p21* and *PUMA*, which can lead to cell growth inhibition or apoptosis^37^. As shown in Fig. 3c, loss of *SMG7* abrogated p53 stabilization and p21 induction, but these responses to Doxo treatment remained largely intact in the K66E mutant cells. Moreover, we found no major defect in induction of p21 in the K66E mutant cells treated with CPT (Supplementary Fig. S4c) and p53 Ser15 phosphorylation – a p53 activation modification^38^ – was not affected by the K66E mutation (Supplementary Fig. S4a,c), suggesting that DNA damage-induced SMG7 binding is not required for p53 activation. Then, we performed cell labeling using the thymidine analog CldU to measure cell proliferation. Unlike *SMG7* KO cells, which retain a significant proliferating population (~15%) as indicated by positive CldU staining, both wild type and *SMG7* K66E mutant cells nearly ceased proliferation following treatment with Doxo (only 2–5%) (Fig. 3d,e). These results clearly demonstrate that the *SMG7* KO cells have a major defect in the p53-mediated cell growth arrest following DNA damage, but the K66E mutant cells are only slightly different from wild type cells.

In an attempt to assess the role of SMG7 binding in regulation of p53 more thoroughly, we further examined p53-mediated apoptosis in response to DNA damage. Notably, CPT treatment strongly induced PUMA expression and apoptosis (as indicated by cleavage of Caspase3 and Parp)^39,40^ in a time-dependent manner; however, we found no major differences between control and *SMG7* K66E mutant cell lines (Fig. 3f). In line with recent studies suggesting that NMD factors such as UPF1 may undergo caspase-dependent cleavage during apoptosis^41,42^, we also observed progressive reduction in the protein levels of wild type and K66E mutant SMG7 following treatment with CPT (Fig. 3f). The results suggest that disruption of SMG7’s 14-3-3 conserved residue lysine 66 and its interaction with p53 has minimal effect on p53-mediated apoptosis.

Taken together, these data suggest that DNA damage-induced SMG7 interaction with p53 is dispensable for p53-mediated gene activation (e.g., *p21* and *PUMA*) and stress response including cell cycle arrest and apoptosis.

### K66E knockin cells retain robust NMD function in the absence of SMG7-UPF1 binding

**Figure 4:**
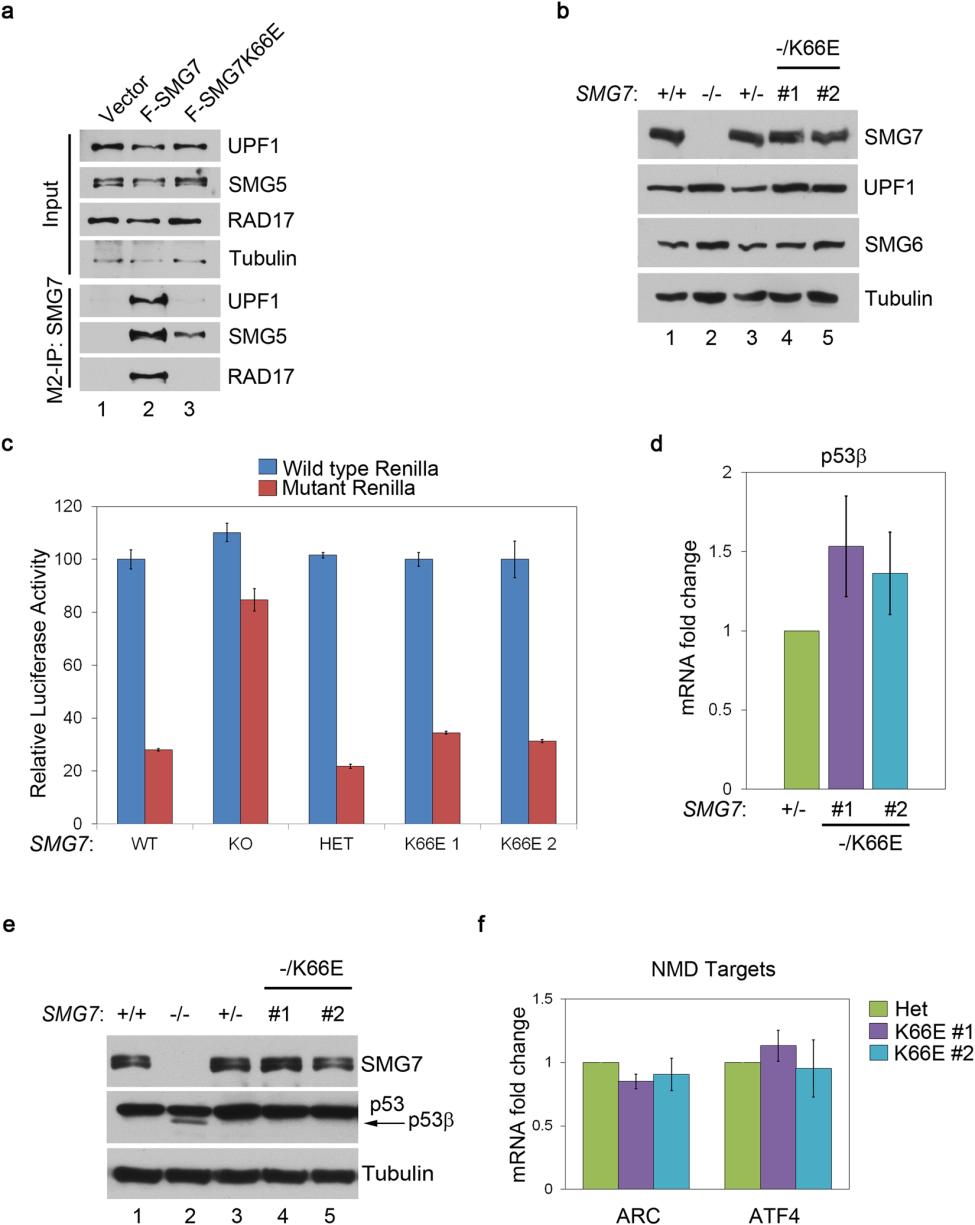
K66E knockin cells retain robust NMD function in the absence of SMG7-UPF1 binding. (**a**) Cell extracts and α-Flag (SMG7) immunoprecipitates from cells expressing F-SMG7 or F-SMG7-K66E were analyzed by western blot using antibodies as indicated. (**b**) Western blot analysis of key NMD factors UPF1 and SMG6 in HCT116 cell lines with various *SMG7* genotypes. (**c**) Assessment of NMD activities using luciferase reporter assay (see Methods). Persistence of luciferase indicates lack of NMD. (**d**) qPCR analysis of p53β expression in control and K66E mutant cells (n = 7, p > 0.2; error bars represent SEM). (**e**) Western blot analysis of p53β protein levels using p53 antibody DO1. The p53β band (indicated by arrow) is slightly below p53α. (**f**) qPCR analysis of expression of two endogenous NMD targets ARC and ATF4 in control and K66E mutant cells. (n = 6, p > 0.3; error bars represent SEM).

In addition to regulating p53 stability, SMG7 controls level of the p53β transcript, a p53 splice isoform degraded by NMD^25^. Through binding to S1096-phosphorylated UPF1, SMG7 regulates UPF1 phosphorylation status by recruiting phosphatase PP2A, which appears to be an indispensable regulatory element for fully functional NMD^7^. Although a recent study suggests that hyperphosphorylation of UPF1 on S1096 and other serine or threonine residues plays an important role in NMD, it not clear whether phosphoserine-mediated SMG7 binding is required for UPF1-dependent NMD^16^. To address this issue, we first assessed the interaction between UPF1 and SMG7 K66E mutant. As expected, SMG7 K66E fails to interact with UPF1 but retains its ability to bind SMG5 (Fig. 4a, lane 2 vs 3), validating that K66E mutation is sufficient to disrupt SMG7 interaction with UPF1 without affecting SMG7-SMG5 dimerization. Assessment of several key NMD factors including UPF1 and SMG6 showed that their protein levels were similar between the control and mutant cell lines, suggesting that core elements of NMD in the K66E mutant cells are largely intact except the SMG7-UPF1 interaction (Fig. 4b).

To determine the role of SMG7-UPF1 binding in NMD, we utilized a previously reported PTC-mediated luciferase reporter assay^43^, which has been used by many groups to establish NMD functionality. Notably, as opposed to *SMG7* KO cells, the K66E mutant cell lines exhibited strong NMD activities similar to wild type cells (Fig. 4c). To corroborate these findings, we also examined expression of p53β – a p53 splice transcript containing PTC^25^ and found that it was not upregulated at protein or mRNA levels in the K66E mutant cells, compared with *SMG7* KO cells (Fig. 4d,e). These results suggest that SMG7-K66E is fully functional in degradation of PTC-containing NMD targets. In addition to PTC, transcripts containing other cis elements including long 3′ untranslated region (UTR) or upstream open reading frame (uORF) are likely subject to NMD-mediated degradation^44,45^. To test whether SMG7 K66E regulates targets in these categories, we examined ARC (long 3′ UTR) and ATF4 (uORF), and observed no significant differences in their transcript levels between control and mutant cells (Fig. 4f). Thus, our data suggest that phosphoserine-mediated SMG7 interaction with UPF1 is not completely required for NMD, at least in our current experimental system.

## Discussion

SMG7 is a 14-3-3-related protein that plays critical roles in regulating p53-dependent DNA damage response and UPF1-mediated NMD^8,9,19^. Here, we investigated the DNA damage-induced interaction of SMG7 with p53, and determined its role in p53 stabilization and activation. Our finding that the 14-3-3-like domain of SMG7 mediates the interaction with phosphorylated Ser15 of p53 demonstrates SMG7s function as phosphoserine-binding protein in a similar mode as conventional 14-3-3 proteins (Figs 1 and 2). However, after examining several SMG7 client proteins, UPF1, p53 and RAD17, we discovered that they share a previously unappreciated conserved binding motif that is completely distinct from the conventional 14-3-3-binding sites (Fig. 1d)^2^. The SMG7-binding motif appears to contain an invariant LSQ sequence, which may serve as a phosphorylation site for members of the phosphoinositide 3-kinase related kinase family such as ATM, ATR and SMG1^46,47^. The fact that all three kinases phosphorylate the SQ motif of SMG7 client proteins suggests specific roles of SMG7 in cellular functions involving ATM, ATR and SMG1, such as DNA damage response and NMD. It is also important to note that SMG7 may only interact with a subset of SQ-containing substrate proteins, given the sequence stringency of SMG7-binding motif. Nonetheless, identification of additional SMG7 client proteins will help define the SMG7-binding motif and elucidate its role in cell signaling pathways.

In assessing the role of phosphoserine-mediated SMG7 binding, we generated SMG7 K66E-knockin cell lines to examine p53- and UPF1-dependent functions. Unexpectedly, our data show that loss of SMG7 binding had no impact on p53 stabilization/activation, or p53-mediated cellular response to DNA damage (Fig. 3). These results are in contrast to what we observed in *SMG7* KO cells, suggesting that SMG7 K66E mutant fully retains its ability to regulate p53 though other mechanisms. In support of this notion, we found that IR induced normal MDM2 Ser395 phosphorylation – a critical mechanism for SMG7 regulation of p53 stability – in the K66E mutant cells (Fig. 3b), suggesting that SMG7-K66E can still inhibit MDM2 (Fig. 3g), which is consistent with the result that SMG7-K66E mutant is able to bind MDM2 (Supplemental Fig. S4d). Furthermore, consistent with our *in vitro* data (Fig. 2a,b), other regions of SMG7 could facilitate a minor interaction with p53 as is observed under basal conditions which may provide additional functional regulation. A possible mechanism could be suggested by our previous GST findings^19^ showing that SMG7 can also interact with the C-terminal regulatory domain of p53. This interaction might contribute to the p53 stabilization phenotype observed by masking the ubiquitination sites present on the p53 regulatory domain. Further exploration of the other p53 interactions with SMG7 *in vivo* and investigation of the regulation of MDM2 could provide further insight into the specific mechanisms for SMG7 regulation of p53.

Also to our surprise, the K66E mutant cells failed to exhibit major defects in NMD (Fig. 4), given that phosphorylation-dependent SMG7-UPF1 binding has been thought to be important for UPF1-mediated NMD^12,48^. Previous studies report SMG7 mainly binds phosphorylated S1096 of UPF1, and the S1096A mutation abolishes UPF1-SMG7 interaction^14^. Thus, our findings that the K66E mutant cells retain robust NMD function in the absence of UPF1 binding suggest that the SMG7 interaction with S1096 phosphorylation of UPF1 might not be as critical as previously thought. Furthermore, our data are consistent with the findings from a recent study suggesting that phosphorylation of each individual site on UPF1 appears to be nonessential for NMD, although hyperphosphorylation of UPF1 on multiple sites is critical for the full NMD function^16^.

It is important to note that our data does not completely rule out the possibility that phosphoserine-mediated SMG7 interaction with UPF1 may play a more prominent role in NMD under certain conditions. For example, we focused primarily on PTC-mediated NMD using the reporter assay and p53β mRNA, and did not extensively examine NMD targets in other categories such as long 3′ UTR and uORF. It is possible that certain NMD targets containing these cis elements will be subject to regulation by phosphoserine-mediated SMG7/UPF binding in a more significant manner. In addition, there could be a tissue-specific role for phosphoserine-mediated SMG7/UPF1 binding in NMD, which we failed to identify in our present study, as we examined NMD specifically in the human colon cancer HCT116 cells.

In summary, our present study revealed some surprising and counterintuitive findings regarding the role of the 14-3-3-related protein SMG7 in regulation of its client proteins p53 and UPF1. Future studies to identify additional phosphorylation-dependent and -independent functions of SMG7s interaction with p53 and UPF1 can provide insight into the novel function of this 14-3-3 like domain and the pathways it regulates.

## Methods

### Cell culture and transfection

HCT116 cells (wild type; gifted by Dr. Bert Vogelstein) and HCT116 *SMG7*^*−/−*^ cell lines were generated in our previous study^19^. HCT116 WT and derivative cell lines were all maintained in McCoy’s 5 A Medium (Cellgro) supplemented with 10% fetal bovine serum (Sigma). U2OS, H1299 (p53 null) cells were maintained in DMEM (Cellgro) supplemented with 10% fetal bovine serum (Sigma). Transfections with plasmid DNA were performed using Lipofectamine 2000 (Invitrogen, Carlsbad, CA, USA) according to the manufacturer’s protocol.

### Treatment conditions

Cells treated with IR were treated in the Gamma Cell irradiator for the necessary time for correct dosage (10 GY) and incubation times begin at the completion of irradiation. DNA damage drugs: Etoposide (20 µM, Sigma), Doxorubicin (0.4 µM, Sigma), Camptothecin (1 µM, Sigma), ATM inhibitor (KU55933, 10 µM 1 hour pre-incubation; control treated with DMSO, Selleckchem), Caffeine (ATM inhibitor, Sigma, C0750) lambda protein phosphatase and buffer (treated according to the manufacturer protocol, treatment following immunoprecipitation and prior to elution, NEB, P0753). Clonal selection, treatment with neomycin (Geneticin (G418) 400 µg/ml, Thermo Fisher).

### CldU staining

Cells were plated on poly-lysine coated coverslips and allowed to adhere for 24–48 hours. Cell were then treated with 1 µm campothecin for indicated times, and CldU (Fisher Scientific) was added to the medium at a final concentration of 25 μm, Including no treatment controls. After incubation with CldU for 1 h, cells were fixed with 70% ethanol at room temperature for 20 minutes. Next the samples were denatured with 3 M HCL and allowed to shake for 30 minutes at room temperature. Samples were washed with PBS and blocked with 3% BSA prepared in PBST and allowed to shake for 30 minutes at room temperature. Samples were incubated overnight on a shaker at 4 °C with primary antibody (Rat Anti- BrdU, BioRad). Following a wash with PBST, samples were incubated with secondary antibody (Alexa Fluor 488 goat anti-Rat IgG, Thermo Fisher) covered at room temperature on the shaker for 30 minutes. Samples were washed and stained for 5 minutes with DAPI (1 µg/ml water, Sigma) on the shaker. Coverslips were then mounted to slides for visualization using mounting media (ProLong Diamond Anti-fade mountant, Thermo Fisher). Slides were imaged on Olympus BX61 upright fluorescent microscope and images were analyzed using ImageJ.

### Generation of SMG7 mutant cell lines HCT116 SMG7^−/+^, SMG7^−/K66E^

pAAV Sept Vector was utilized to target exon 4 of *SMG7* transcript (Lysine 66 residue). The left homology arm contained the knock-in point mutation (substitution of G for A, changing lysine to glutamic acid). See targeting scheme and primer chart (Supplementary Fig. 3) for additional information. Primers P2-P4 were utilized to amplify the left homology arm an introduce the K66E mutation. Primers P5 & p6 were utilized to amplify the right homology arm. Both arms were amplified, purified and cloned into the pAAV targeting vector to generate pAAV SEPT *SMG7 K66E*. Virus was generated by transfection of the pAAV Sept *SMG7* K66E construct into 293FT cells and harvested via freeze/thaw lysis following 1-day incubation. pAAV Sept *SMG7* K66E virus was used to infect healthy HCT116 cells and following 1-day incubation split into 96 well plates with 400 µg/ml concentration of G418 for selection. Clones were grown up and genotyped (P1 & P7) for integration of the neo cassette into the *SMG7* locus. P1 ensures targeting to the SMG7 locus as it is outside the targeting region, p7 ensures homologous recombination as it was introduced at the end of the left homology arm (LHA). PCR conditions favored PCR products smaller than 2 kb and as such amplified a single band at 1.5 kb. A correct clone was then infected with Ad-Cre virus (MOI 10) to facilitate the removal of the neomycin selection cassette. Following 1-day incubation the pool was serially diluted into 96 well plates and resulting clones were split to identify G418 sensitive clones and confirmed via genotyping (P1 & P8) for the neomycin cassette. P8 is located within the cassette and paired with p1 allows for SMG7 locus specific detection of the selection cassette (2.5 kb). Absence of amplification together with sensitivity to G418 confirms the loss of the selection cassette. Following identification of *SMG7* K66E/*SMG7* WT neomycin- clones, one was chosen for a second round of targeting with pAAV *SMG7* KO virus generated in our previous study^19^. PAAV Sept Vector was utilized to target exon 3 of *SMG7* transcript. The *SMG7* K66E/*SMG7* WT neomycin- clones were infected with the virus and selected under G418 conditions. Clones were grown up and genotyped (P7 & P9) for integration of the neo cassette into the SMG7 locus. A correct clone was identified based on the presence of residual loxP and cloning sites in both mutated alleles resulting in three possibilities. No amplification would indicate the cells were K66E neo^−^ at targeting and the integration of the neomycin cassette occurred somewhere other than the SMG7 locus. 3.8 kb amplification denotes clones that were targeted for SMG7 KO on a previously un altered allele. 3.0 kb amplification indicates the SMG7 KO integration occurred on the same allele as the K66E mutation. 1.3 kb amplification arises from SMG7 K66E neo^+^ targeting as carried out in the first round of targeting (positive control). Correct clones were considered to be targeted for SMG7 K66E neo^−^ from step 2 while the SMG7 KO from step 3 occurred on the other allele, finally generating one KO and one KI allele. Multiple clones were identified, in order to assess clonal variation, and sequenced to confirm the deletion of SMG7 exon 3 and retention of the K66E mutation on the neo^−^ allele. Additional information available upon request.

### U2OS SMG7 Stable, fragment, mutant, p53 mutant

Cloned SMG7/p53 regions into pGIPZ (Open Biosystems, Addgene) lentivirus. Mutant expressing plasmids were sequenced to confirm. Virus was generated by transfection of the pGIPZ constructs into 293FT cells and harvested the media following 1-day incubation. The U2OS cells were infected with the resulting virus for 1 day. Then the infected pool was selected with puromycin (1 µg/ml media) and resistant clones were allowed to grow up. Following several passages, the cells were screened for expression via western blot.

### Western blot and immunoprecipitation

Protein was isolated from the cells using Flag Lysis Buffer (50 mM Tris-HCl [pH 7.9], 137 mM NaCl, 10 mM NaF, 1 mM EDTA, 1% Triton X-100, 0.2% sarkosyl, 10% glycerol, and fresh proteinase inhibitor cocktail (Sigma), some experiments also phosphatase inhibitor cocktail II, (Krackeler). Samples were resolved using SDS-PAGE gel using indicated antibodies. The antibodies used are as follows: UPF1 (rabbit, Bethyl, #A300-036A); SMG6 (rabbit, Abcam, #AB87539); SMG7 (rabbit, LS Bio #CPR2435; rabbit, Bethyl #A302-107A); β-actin (mouse, Santa Cruz, #SC-47778); p53 (mouse, Santa Cruz, DO1 #SC-126); p53 FL (rabbit, Santa Cruz, #SC-6243); anti-pS15-p53 (rabbit, Cell Signaling, 9284,); SMG5 (rabbit, Proteintech, #12694-1-P); Flag (rabbit, Sigma-Aldrich, #F7425); HA (mouse, Covance, #16B12), Caspase 3 (rabbit, Cell Signaling, #9661), puma (rabbit, Upstate, Lake Placid); anti-pS395-Mdm2 (rabbit, Thermo Scientific, PA5-13008); Anti-p21 (rabbit, Santa Cruz, C-19 sc-397); Parp (rabbit, Cell Signaling, 9542S); anti-Mdm2 antibody (mouse, Calbiochem, 4B11 #OP143); P-Mdm2 (ser395) (rabbit, ThermoFisher, #PA5-13008), P-1981 ATM (rabbit, Cell Signaling, D6H9 #5883); 14-3-3σ (mouse, Santa Cruz, #sc-166473), Tubulin (rat, Santa Cruz, #sc-53029). Secondary antibodies: Anti-Rabbit IgG (goat, Cell Signaling, #7074); Anti-Rat IgG (goat, Southern Biotech, #3050-05); Anti-mouse IgG (sheep, GE Healthcare, #NA931); M2 IP (mouse, Sigma, #A2220); 1801 IP (mouse, Santa Cruz, #SC-98-AC).

### qRT PCR

Total RNA was extracted from cells using the TRIzoI reagent (Invitrogen, 15596-026) according to the manufacturer’s protocol. After reverse transcription (MuLV, NEB, M0253), the quantitative PCR was performed in triplicate with SYBR Green Master Mix (Applied Biosystems) and the StepOnePlus Real-Time PCR System (Applied Biosystems) with the following PCR conditions: 10 min. at 95 °C followed by 40 cycles of 95 °C for 15 sec. and 60 °C for 1 min. Primers used for PCR: β-actin (5′-CCAACCGCGAGAAGATGACC-3′ and 5′-CGTTGGCACAGCCTGGATAGCAACG-3′ (14)); p53β (5′-GAGCACTAAGCGAGCACTGCC-3′ and 5′-TTGAAAGCTGGTCTGGTCCTGA-3′ (20)), ATF3 (5′-GCCATTGGAGAGCTGTCTTC-3′ and 5′-GGGCCATCTGGAACATAAGA-3′), ATF4 (5′-GACGGAGCGCTTTCCTCTT-3′ and 5′-TCCACAAAATGGACGCTCAC-3′), ARC (5′-AGCGGGACCTGTACCAGAC-3′ and 5′-GCAGGAAACGCTTGAGCTTG-3′).

### NMD luciferase reporter assays

Constructs were obtained from Andreas Kulozik as a generous gift; pCL-Neo β-globin WT Renilla, pCL- Neo β-globin NT39 Mutant Renilla, and pCL- Neo Firefly^43^. Constructs were all transfected (Lipofectamine 2000, Invitrogen, 11668030) with the *Firefly* control construct (and *GFP* to monitor transfection efficiency), half were also transfected with *Renilla β-globin WT* and half were also transfected with *Renilla β-globin NT39 Mutant*. The cells were harvested after 1.5 days for luciferase detection. Luciferase was detected using a (20/20^n^ Luminomenter, Turner Biosystems) and (Dual Luciferase Reporter Assay System, Promega, E1910). *Renilla* signals were normalized to *firefly* controls.

### GST proteins

Purified SMG7 (fragments: full length, 1-430aa, 431–623aa, 624–821aa, 815–1091aa, 1–821aa, & 431–1091aa; mutant: K66E and K66E/R163E; flag tagged), and GST p53 proteins, as indicated, were incubated with 10 μl GST beads bound with 2 μg of GST or GST-fusion proteins in binding buffer BC100 in the presence of 1 μg μl −1 of BSA on a rotator overnight at 4 °C. The GST beads were washed five times with binding buffer and the bound proteins were eluted by boiling in SDS sample, resolved on an 8% SDS-PAGE and analyzed with western blot analysis using anti-SMG7, p53 antibody.

### Statistical analysis

Microsoft Excel software was used for statistical analysis. Student’s *t*-test was used for comparing two samples (p < 0.05, using a two tailed test). Error bars are utilized to represent SEM.

## Supplementary information

Characterization of SMG7 14-3-3-like domain reveals phosphoserine binding-independent regulation of p53 and UPF1
