# Supplementary material for: Characterization of SMG7 14-3-3-like domain reveals phosphoserine binding-independent regulation of p53 and UPF1
Source: Sci Rep. 2019 Sep 11;9:13097. doi: 10.1038/s41598-019-49229-3 (PMC6739308; doi:10.1038/s41598-019-49229-3)
Supplement: Supplementary file 1 — Characterization of SMG7 14-3-3-like domain reveals phosphoserine binding-independent regulation of p53 and UPF1 [file 41598_2019_49229_MOESM1_ESM.pdf]

Characterization of SMG7 14-3-3-like domain reveals phosphoserine binding-independent regulation of p53 and UPF1

Lauren E. Cowen[1], Hongwei Luo[1] and Yi Tang[1]*

Affiliations: [1]Department of Regenerative and Cancer Cell Biology, Albany Medical College, 47 New Scotland Ave. Albany, NY 12208, USA

*Corresponding Author: tangy@amc.edu

Supplemental Information and Raw Data

SUPPORTING INFORMATION

Supplemental Figure 1: Treatment with various DNA-damaging agents induces interaction of SMG7 with p53

(a) Cell extracts from control or irradiated cells (with increasing doses of gamma irradiation 0.5, 1, 2, 5, and 10 GY) and the α-Flag M2-IP (p53) immunoprecipitates were analyzed by western blot using antibodies as indicated. **(b)** Cells were treated with gamma-radiation (10 Gy, 1 hour), doxorubicin (0.4 μM, 6 hours) or Nutlin (10 μM, 4 hours) to stabilize p53. Cell extracts and the α-Flag (p53) immunoprecipitates were analyzed as in **a**. **(c)** Cell were treated with etoposide (20 μM, 4 hours) in the absence or presence of ATM inhibitor (10 μM) and analyzed as in **a**. **(d)** Cells treated with etoposide (20 μM, 4 hours) alone or together with ATM inhibitor KU55933 (10 μM) or Caffeine (3 mM, an ATM and ATR inhibitor) were analyzed as in **a**. **(e)** Flag-tagged p53 and untagged SMG7 were expressed in H1299 cells. The α-Flag M2-IP (p53) immunoprecipitates were untreated, or treated with lambda phosphatase buffer alone or lambda phosphatase enzyme and buffer, followed by elution with Flag peptide. The eluted materials were analyzed by western blot using antibodies as indicated.

Supplemental Figure 2: SMG7/SMG5 heterodimer can also interact with p53

(a) Scheme depicting sequence alignment of conserved lysine residues between 14-3-3 and SMG family proteins. Note: red font denotes presence of conserved binding residue (14-3-3ζ & SMG7 (Lysine 66)), blue font denotes alternative residues at the critical site (SMG5 & SMG6). **(b)** Cell extracts and the α-Flag (p53) immunoprecipitates were analyzed by western blot using antibodies as indicated following treatment with 0.5 μM camptothecin for various time points or Nutlin (10 μM for 12 hours). **(c, d)** M2-IP (SMG7 or SMG5) following overexpression of Flag-tagged wild type SMG7/SMG5 and untagged SMG7, SMG5 and SMG7 K66E, together with p53 in H1299 cells. Samples were analyzed by western blot using antibodies as indicated.

Supplemental Figure 3: Generation of SMG7 K66E cell line

(a) Scheme of AAV-generated SMG7$^{-/K66E}$ neo^{+} cell lines. See details of generation of HCT116 SMG7$^{-/K66E}$ neo^{+} and SMG7$^{-/+}$ neo^{+} cells in Methods. **Note:** asterisks represents the K66 residue, black* indicates wild type and red* indicates K66E mutation. **(b)** Genotyping results for confirmation of integration of SMG7 K66E targeting at the correct locus. See Methods for details. **(c)** Final genotyping results to confirm correct clones. Primer pair P7/P9 was utilized as a representative image. WT denotes wild type HCT116 genomic DNA. + denotes the parental clone generated in the first round of targeting (SMG7 K66E neo^{+}); positive amplification control but smaller size. - denotes the parental clone following removal of the neomycin cassette serving as a negative amplification control. Correct clones were considered to be targeted for SMG7 K66E neo^{-} while the SMG7 KO neo^{+} occurred on the other allele, finally generating one KO and one KI allele (Outcome (I)). **(d)** Primer sequences of all primers utilized in the study for either cloning or genotyping. See Methods for details. **(e)** Sequencing results of SMG7 locus modified to generate our three cell lines. HCT116 SMG7: wild type/SMG7 knock-out (KO), SMG7 KO/SMG7 K66E Clone #1, and SMG7 KO/SMG7 K66E Clone #2. Wild type: TTTAAG; K66E Mutant: TTTGAG.

Supplemental Figure 4: SMG7 interaction with p53 not required for p53 stabilization following various forms of DNA damage

(a) Western blot analysis of HCT116 cell lines treated with IR (10 GY for 16 hours). **(b)** Western blot analysis of HCT116 cell lines treated with doxorubicin (0.4 μM for 6 hours). **(c)** Western blot analysis of HCT116 cell lines treated with camptothecin (CPT, 1 μM for 6 hours). **(d)** M2-IP (SMG7) following overexpression of Flag-tagged SMG7 K66E and untagged MDM2 in H1299 cells. Samples were analyzed by western blot using antibodies as indicated.

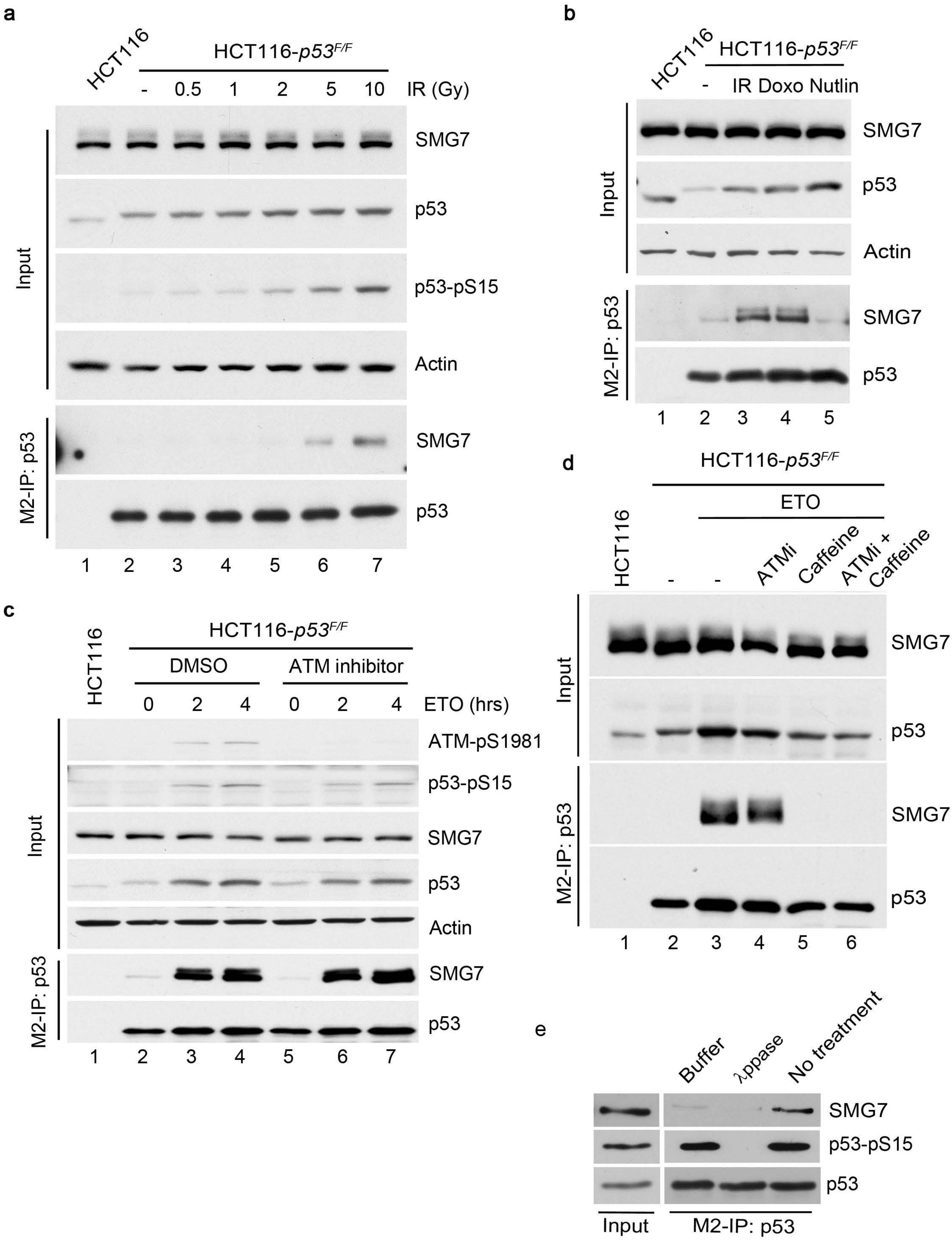
a
HCT116
HCT116-p53F/F
- 0.5 1 2 5 10 IR (Gy)
Input
SMG7
p53
p53-pS15
Actin
M2-IP: p53
SMG7
p53
1 2 3 4 5 6 7
b
HCT116
HCT116-p53F/F
- IR Doxo Nutlin
Input
SMG7
p53
Actin
M2-IP: p53
SMG7
p53
1 2 3 4 5
c
HCT116
HCT116-p53F/F
DMSO
ATM inhibitor
0 2 4 0 2 4 ETO (hrs)
Input
ATM-pS1981
p53-pS15
SMG7
p53
Actin
M2-IP: p53
SMG7
p53
1 2 3 4 5 6 7
d
HCT116-p53F/F
ETO
HCT116
- - ATMi Caffeine ATMi + Caffeine
Input
SMG7
p53
M2-IP: p53
SMG7
p53
1 2 3 4 5 6
e
Buffer λppase No treatment
SMG7
p53-pS15
p53
Input
M2-IP: p53

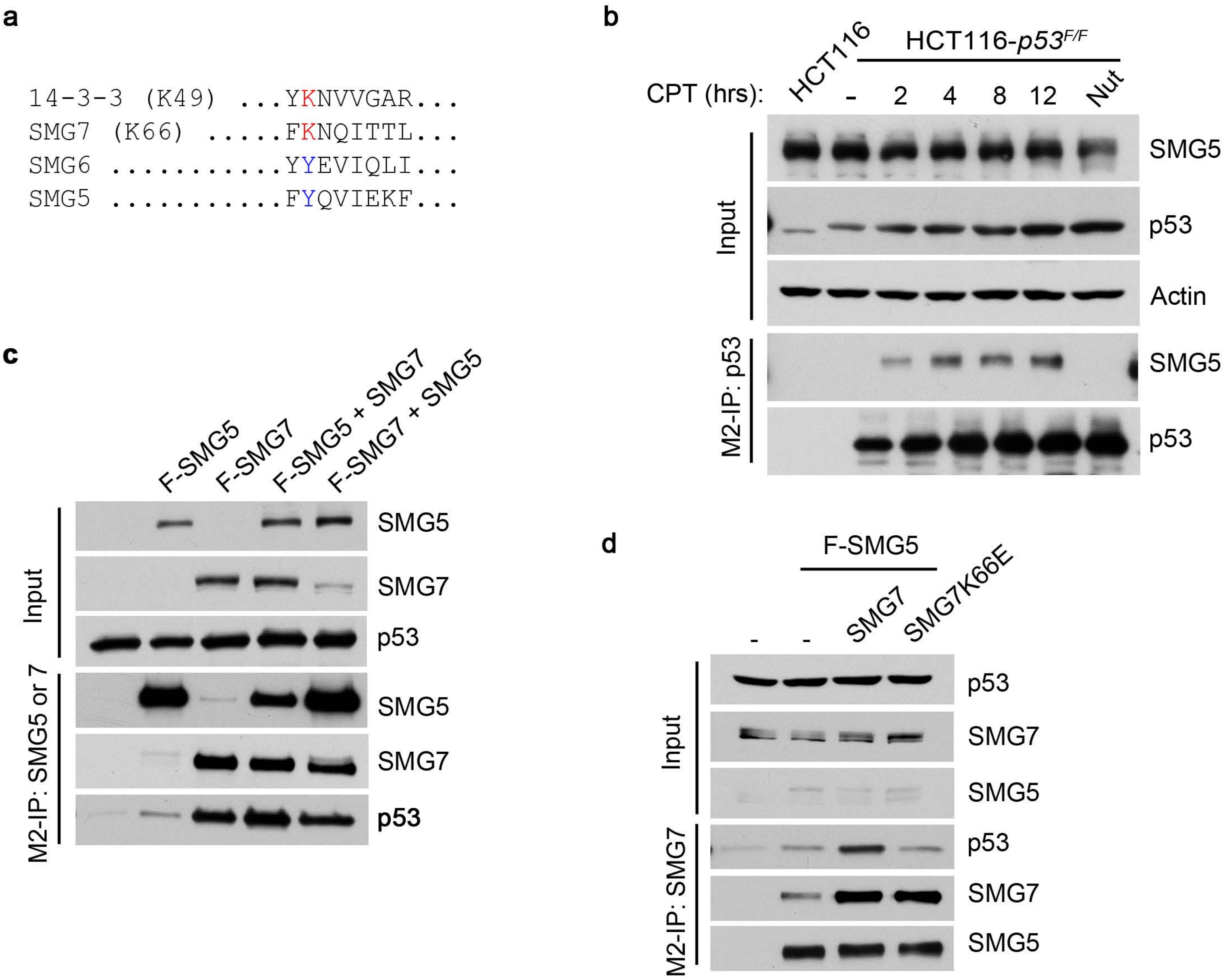
a
14-3-3 (K49) ...YKNVVGAR...
SMG7 (K66) .....FKNQITTL...
SMG6 ....YYEVIQLI...
SMG5 ....FYQVIEKF...
b
HCT116
HCT116-p53F/F
CPT (hrs):
-
2
4
8
12
Nut
Input
M2-IP: p53
SMG5
p53
Actin
SMG5
p53
c
F-SMG5
F-SMG7
F-SMG5 + SMG7
F-SMG7 + SMG5
Input
M2-IP: SMG5 or 7
SMG5
SMG7
p53
SMG5
SMG7
p53
d
F-SMG5
-
-
SMG7
SMG7K66E
Input
M2-IP: SMG7
p53
SMG7
SMG5
p53
SMG7
SMG5

a b

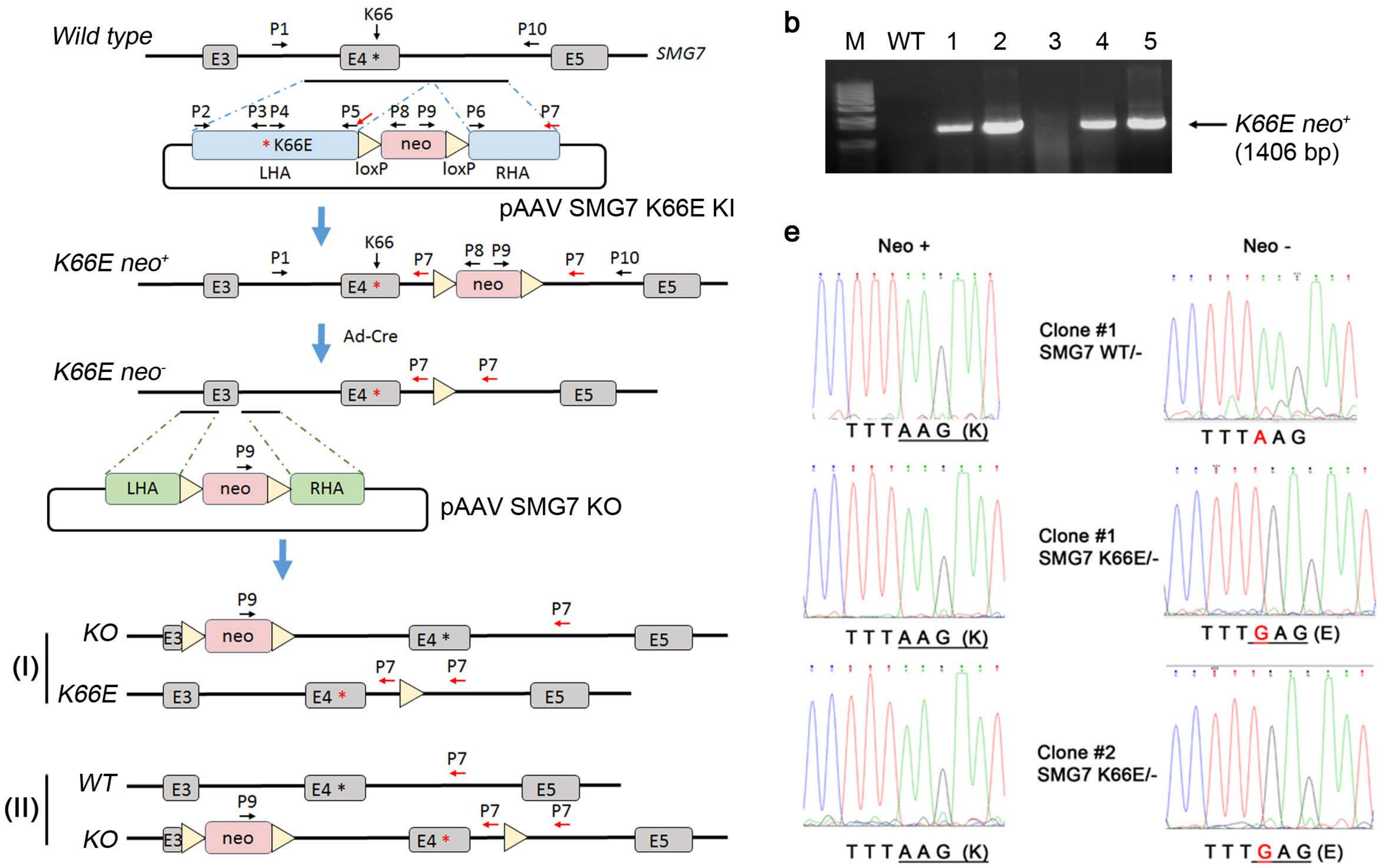

c

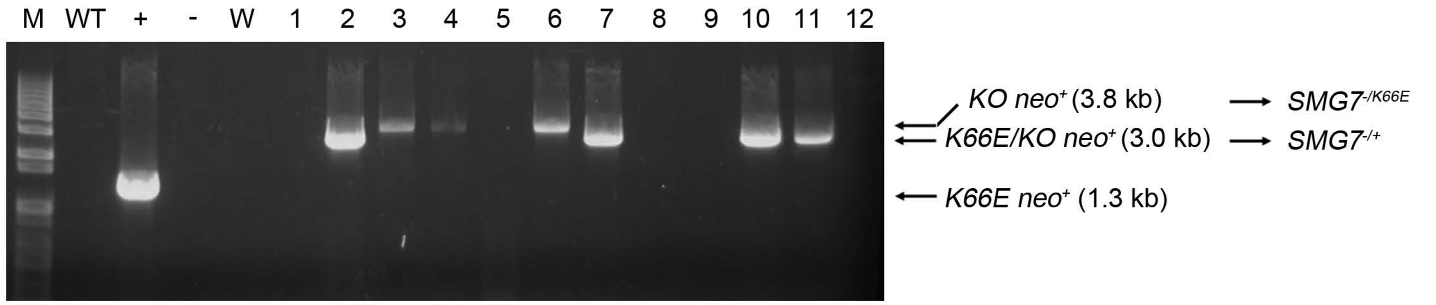

d

Primer Name	Primer Sequence
P1	CACTGTGGTTTCCAGGTGAG
P2	aaaccggtGCAGTGAAACTGAATGCTCTGG
P3	pAAAGGCGTGATTCCAGCTG
P4	pGAGAATCAGATCACAACACTGC
P5	aagagctcTCTGAATTGTTCCAACACAGGAGGCTTTGTGCTCAATTATCC
P6	aaacatatgTAGTGAAACAGTGGAAGTAGTCAG
P7	aactcgagTCTGAATTGTTCCAACACAGG
P8	GCATCAGAGCAGCCGATTGTC
P9	TGTGGCGGACCGCTATCAGG
P10	CCCTCTCTCGAGAAGCATTCCC

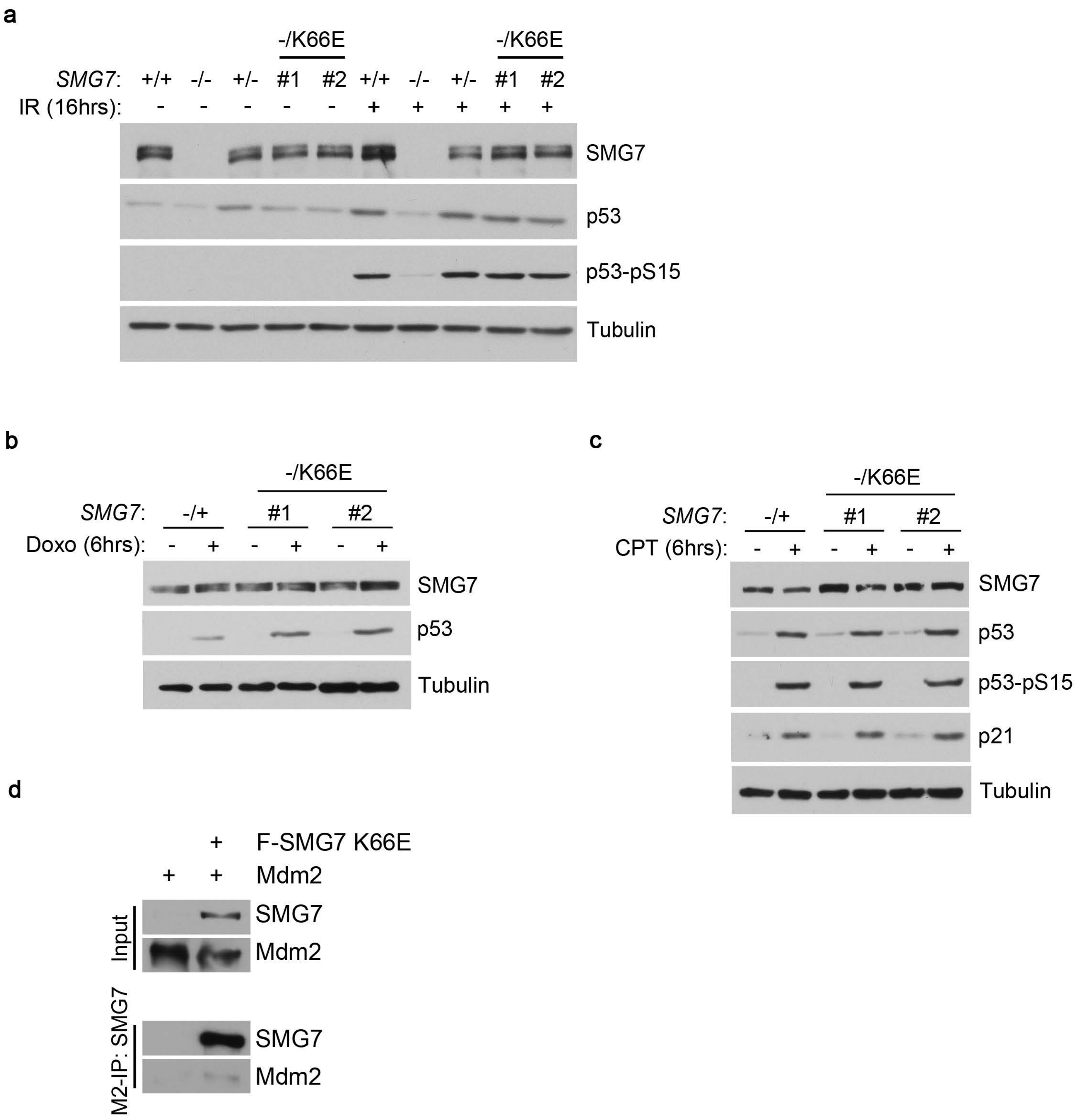
a
-/K66E
-/K66E
SMG7: +/+ -/- +/- #1 #2 +/+ -/- +/- #1 #2
IR (16hrs): - - - - - + + + + +
SMG7
p53
p53-pS15
Tubulin
b
-/K66E
SMG7: -/+ #1 #2
Doxo (6hrs): - + - + - +
SMG7
p53
Tubulin
c
-/K66E
SMG7: -/+ #1 #2
CPT (6hrs): - + - + - +
SMG7
p53
p53-pS15
p21
Tubulin
d
+ F-SMG7 K66E
+ + Mdm2
Input
SMG7
Mdm2
M2-IP: SMG7
SMG7
Mdm2

Figure 1

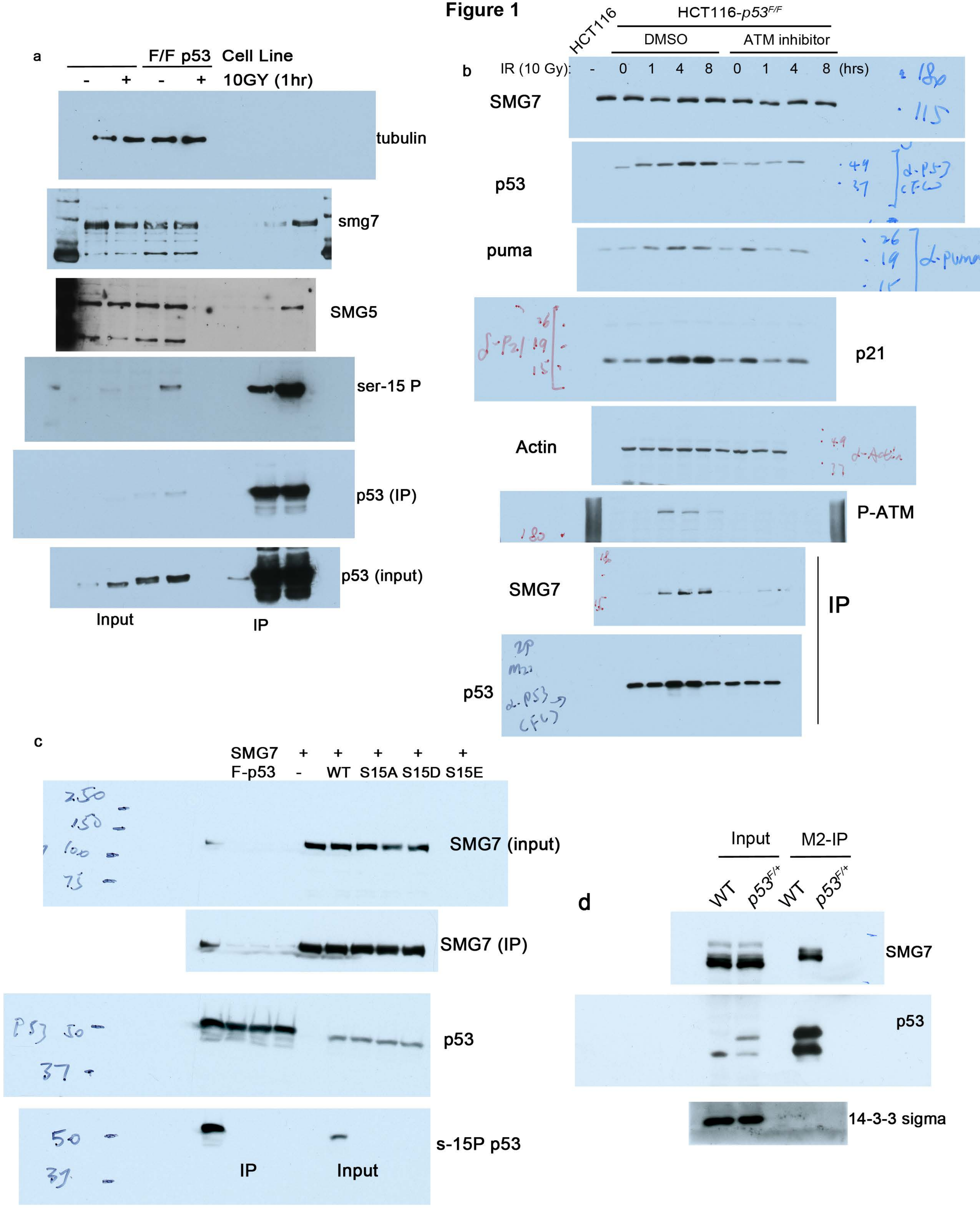

a
F/F p53 Cell Line
- + - + 10GY (1hr)
tubulin
smg7
SMG5
ser-15 P
p53 (IP)
p53 (input)
Input
IP
b
HCT116
HCT116-p53F/F
DMSO
ATM inhibitor
IR (10 Gy): - 0 1 4 8 0 1 4 8 (hrs)
SMG7
p53
puma
p21
Actin
P-ATM
SMG7
p53
IP
c
SMG7 + + + + +
F-p53 - WT S15A S15D S15E
SMG7 (input)
SMG7 (IP)
p53
s-15P p53
IP
Input
d
Input
M2-IP
WT
p53F/+
WT
p53F/+
SMG7
p53
14-3-3 sigma

Figure 2

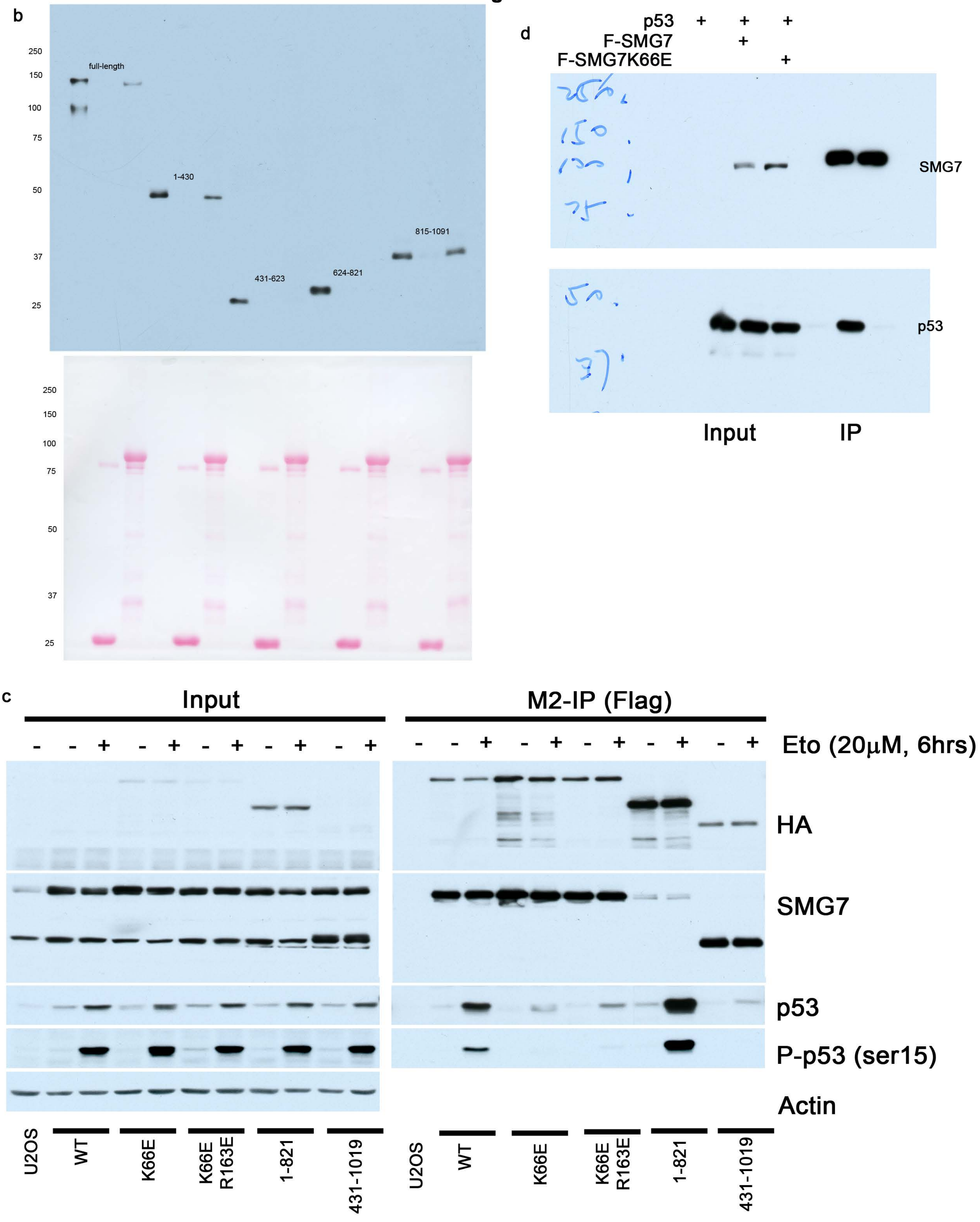
b
250
150
100
75
50
37
25
full-length
1-430
431-623
624-821
815-1091
250
150
100
75
50
37
25
d
p53 + + +
F-SMG7 +
F-SMG7K66E +
SMG7
p53
Input
IP
c
Input
M2-IP (Flag)
Eto (20μM, 6hrs)
HA
SMG7
p53
P-p53 (ser15)
Actin
U2OS
WT
K66E
K66E R163E
1-821
431-1019
U2OS
WT
K66E
K66E R163E
1-821
431-1019

Figure 2 continued

e

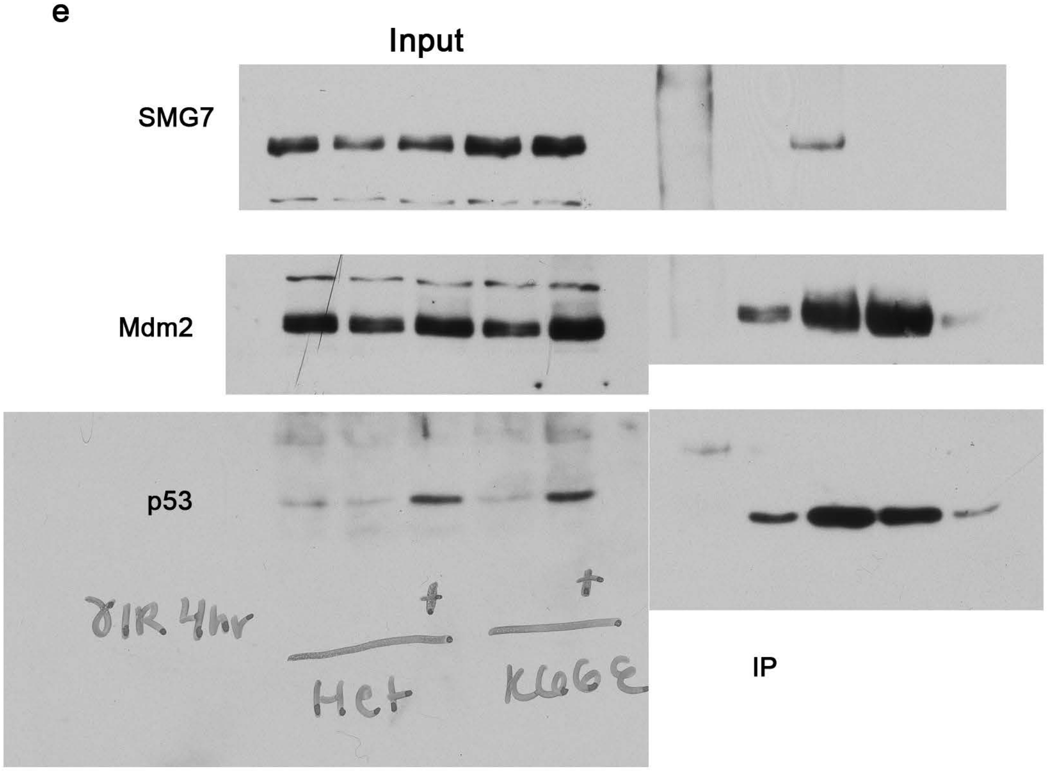

* Note: In IP the 4th and 5th lanes were accidentally reversed

Figure 3

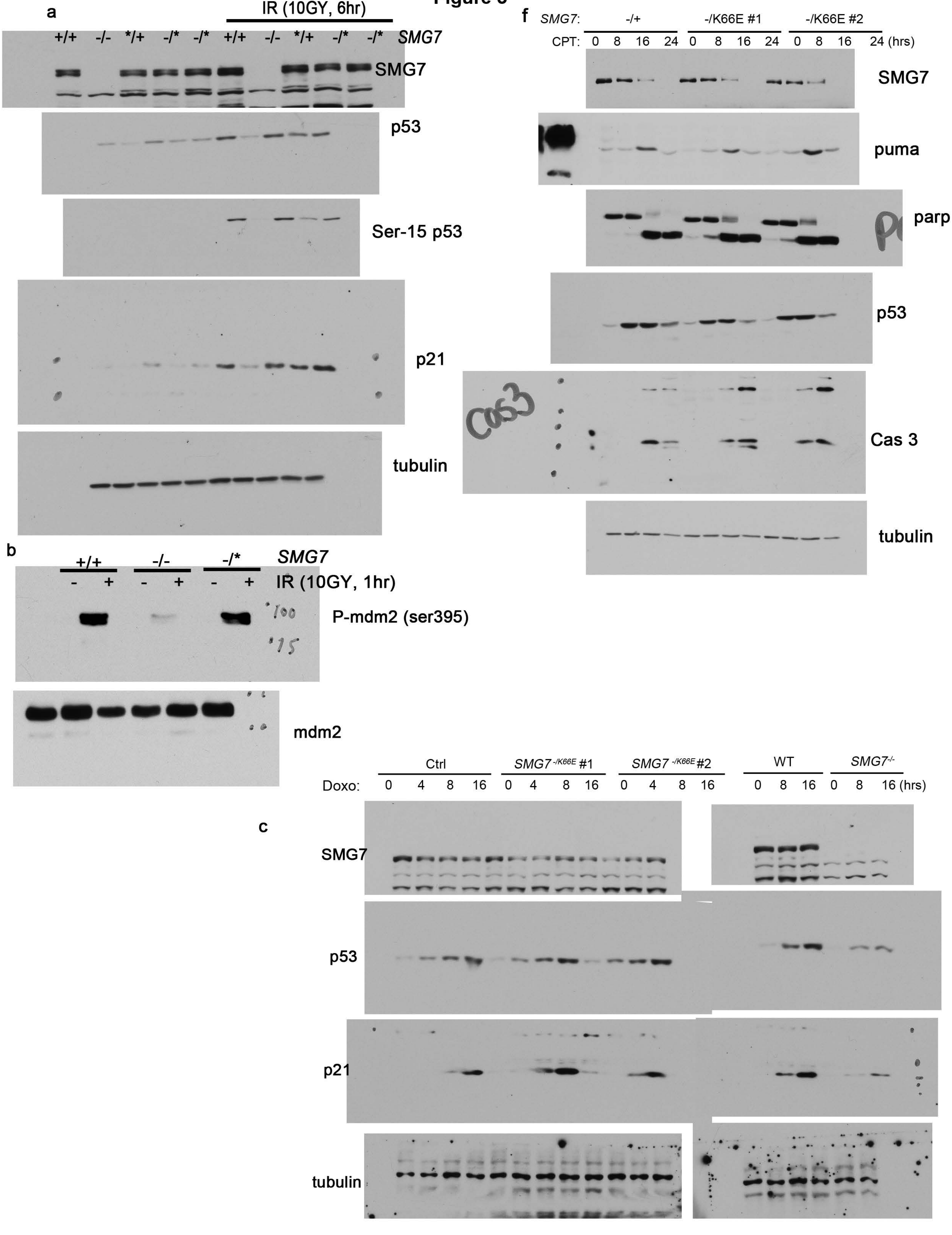
a
IR (10GY, 6hr)
+/+ -/- */+ -/* -/* +/+ -/- */+ -/* -/* SMG7
SMG7
p53
Ser-15 p53
p21
tubulin
b
+/+ -/- -/* SMG7
- + - + - + IR (10GY, 1hr)
P-mdm2 (ser395)
mdm2
f
SMG7: -/+ -/K66E #1 -/K66E #2
CPT: 0 8 16 24 0 8 16 24 0 8 16 24 (hrs)
SMG7
puma
parp
p53
Cas 3
tubulin
c
Ctrl SMG7 -/K66E #1 SMG7 -/K66E #2 WT SMG7-/-
Doxo: 0 4 8 16 0 4 8 16 0 4 8 16 0 8 16 0 8 16 (hrs)
SMG7
p53
p21
tubulin

Figure 4

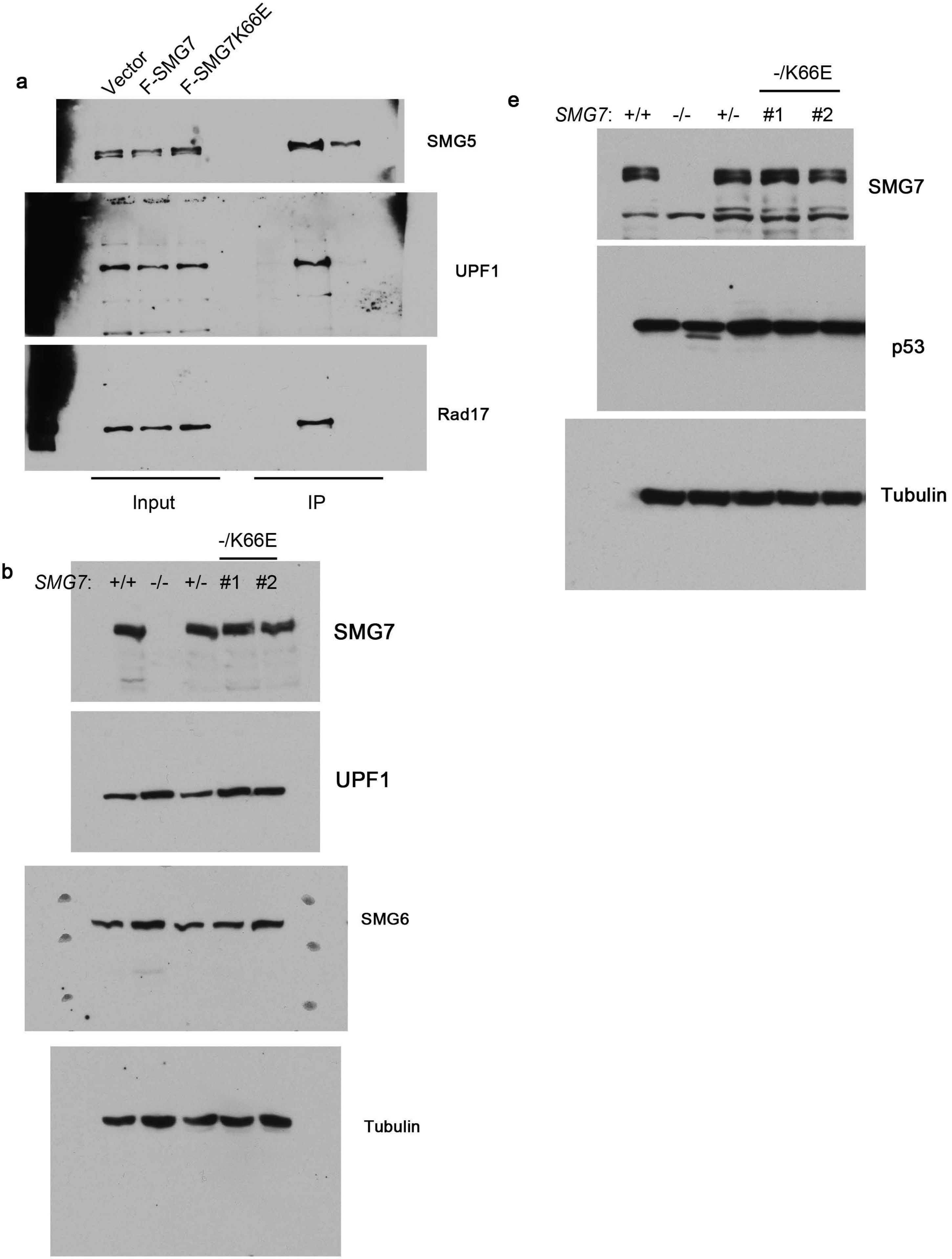
a
Vector
F-SMG7
F-SMG7K66E
SMG5
UPF1
Rad17
Input
IP
b
-/K66E
SMG7: +/+ -/- +/- #1 #2
SMG7
UPF1
SMG6
Tubulin
e
-/K66E
SMG7: +/+ -/- +/- #1 #2
SMG7
p53
Tubulin

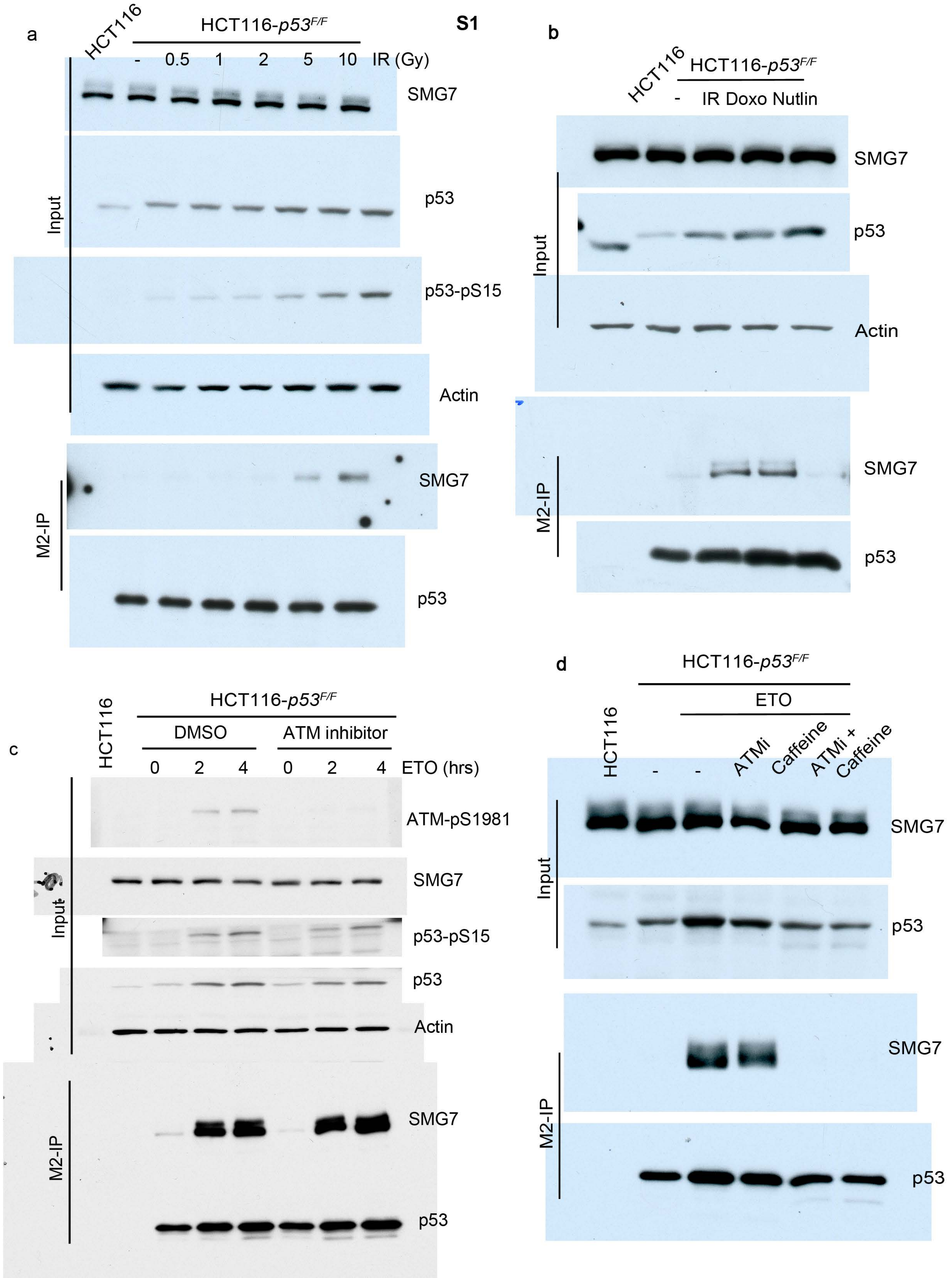
S1
a
HCT116
HCT116-p53F/F
- 0.5 1 2 5 10 IR (Gy)
SMG7
p53
p53-pS15
Actin
Input
SMG7
p53
M2-IP
b
HCT116
HCT116-p53F/F
- IR Doxo Nutlin
SMG7
p53
Actin
Input
SMG7
p53
M2-IP
c
HCT116
HCT116-p53F/F
DMSO
ATM inhibitor
0 2 4 0 2 4 ETO (hrs)
ATM-pS1981
SMG7
p53-pS15
p53
Actin
Input
SMG7
p53
M2-IP
d
HCT116-p53F/F
ETO
HCT116
- - ATMi Caffeine ATMi + Caffeine
SMG7
p53
Input
SMG7
p53
M2-IP

S1 cont.

e

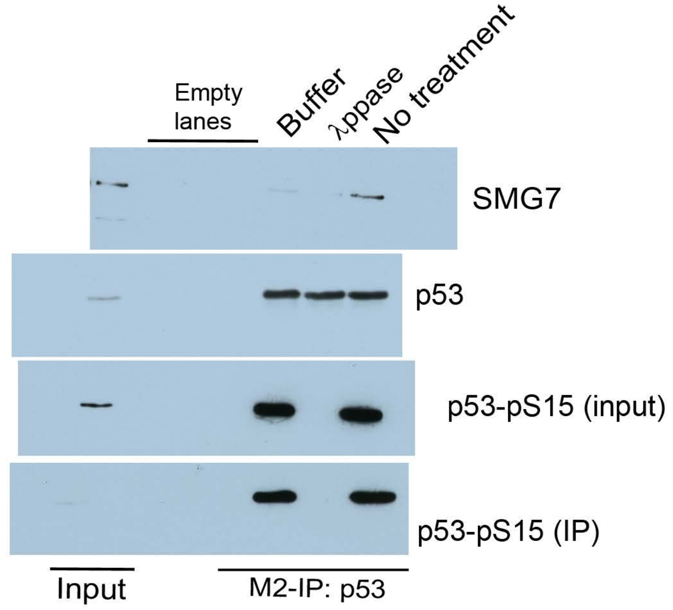

S2

b

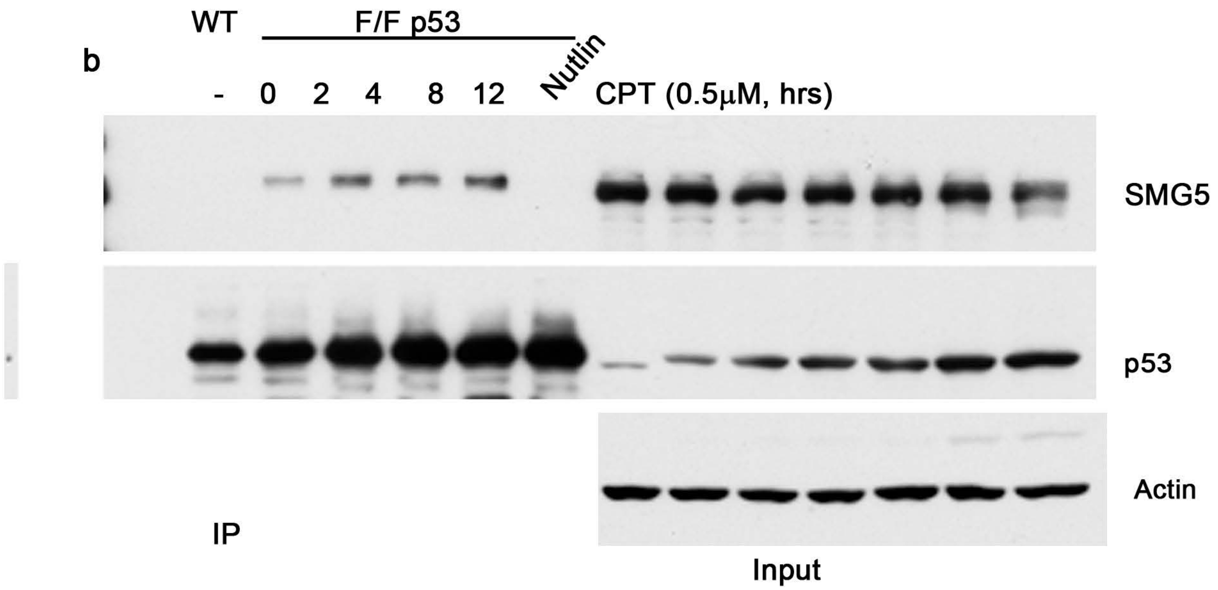

c

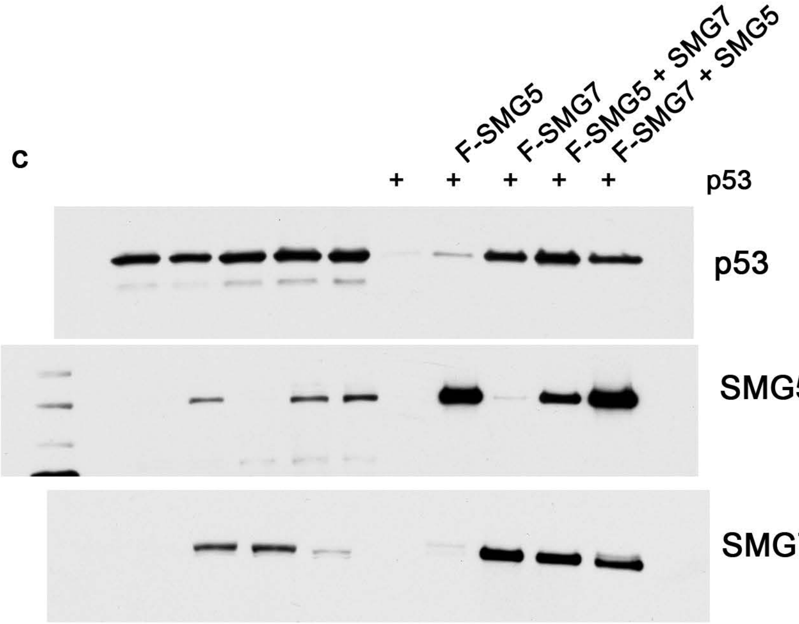

d

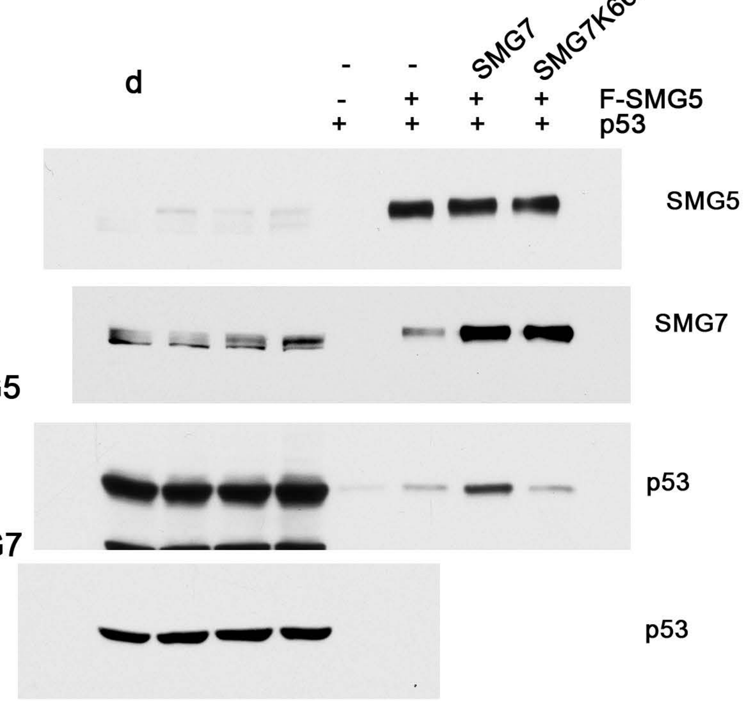

a

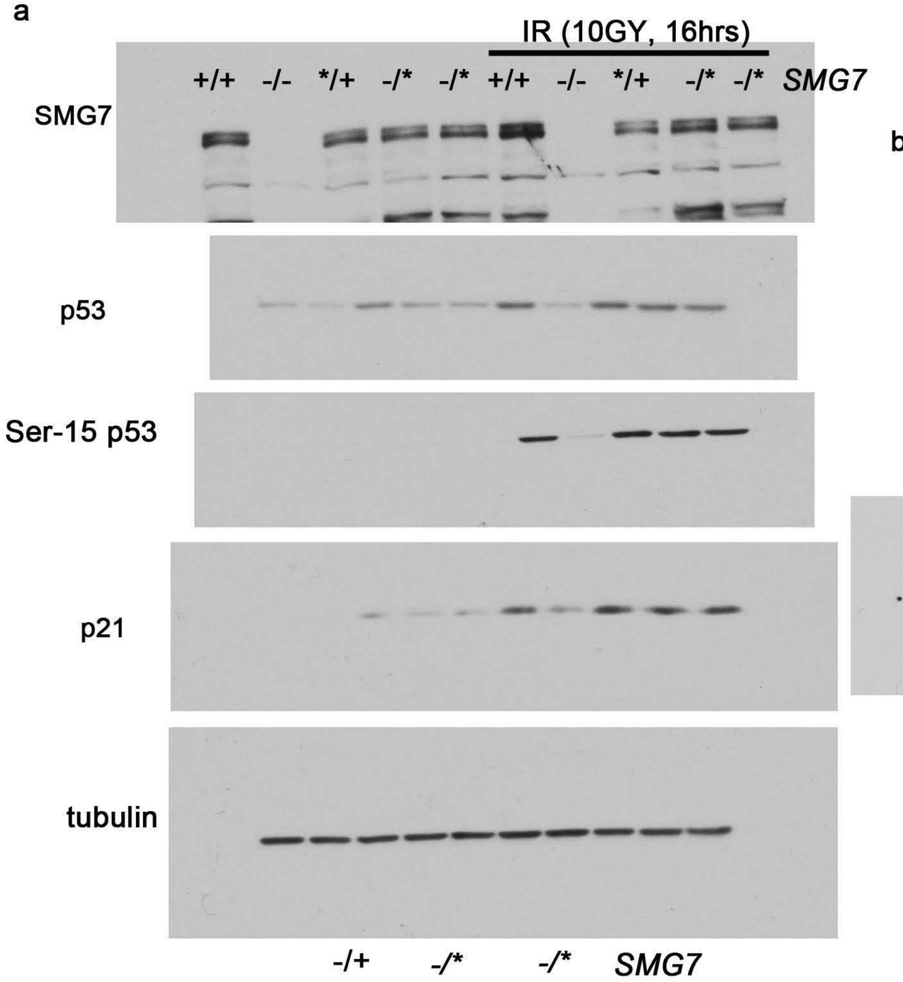
IR (10GY, 16hrs)
+/+ -/- */+ -/* -/* +/+ -/- */+ -/* -/* SMG7
SMG7
p53
Ser-15 p53
p21
tubulin
-/+ -/* -/* SMG7

b

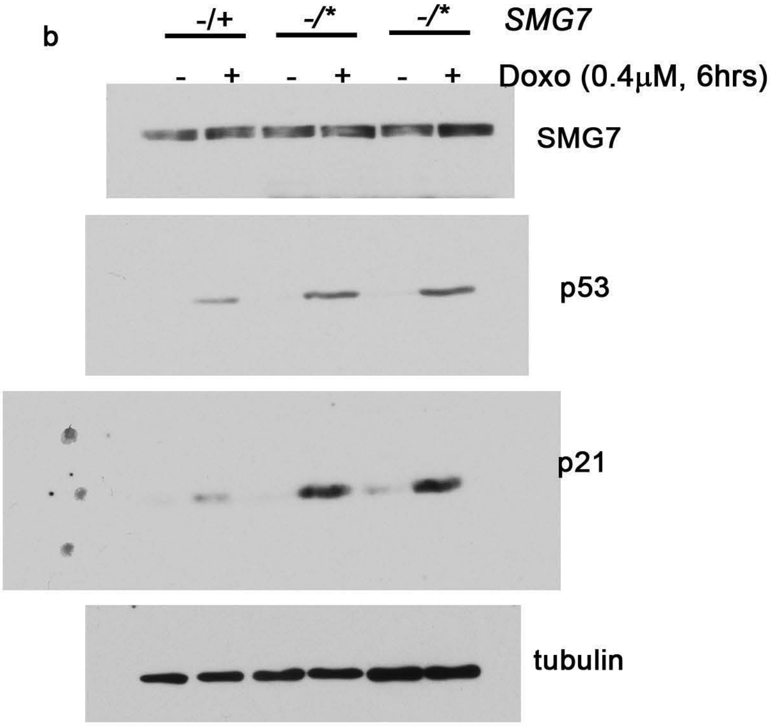
-/+ -/* -/* SMG7
- + - + - + Doxo (0.4μM, 6hrs)
SMG7
p53
p21
tubulin

c

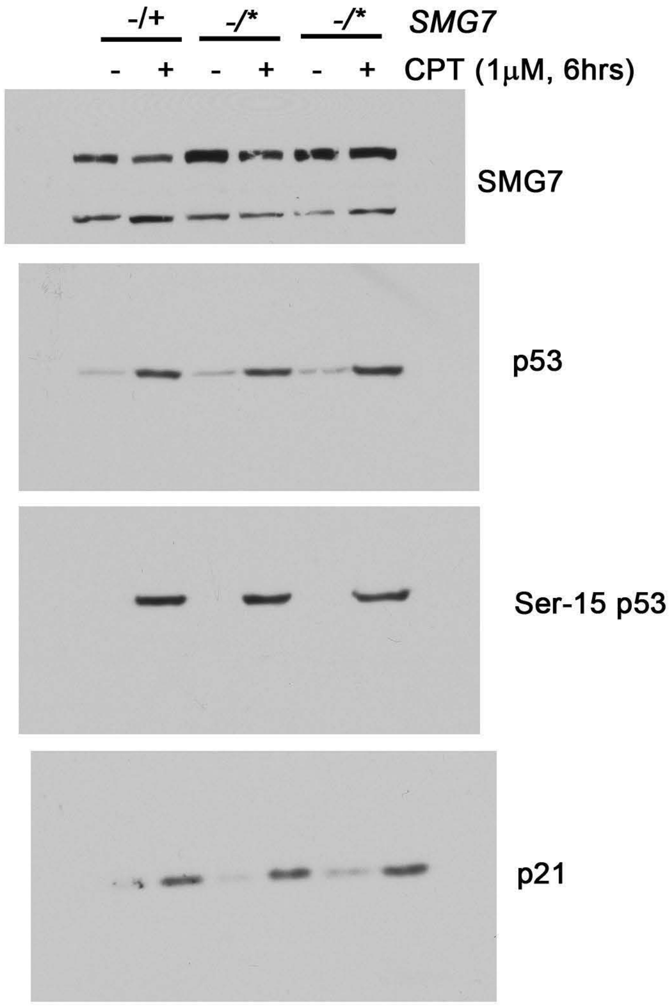
-/+ -/* -/* SMG7
- + - + - + CPT (1μM, 6hrs)
SMG7
p53
Ser-15 p53
p21

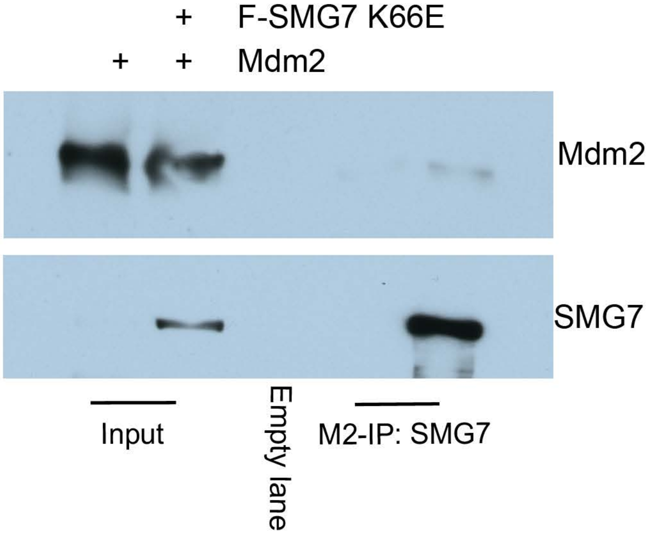
+ F-SMG7 K66E
+ + Mdm2
Mdm2
SMG7
Input
Empty lane
M2-IP: SMG7
